# Global analysis of A-to-I RNA editing reveals association with common disease variants

**DOI:** 10.7717/peerj.4466

**Published:** 2018-03-06

**Authors:** Oscar Franzén, Raili Ermel, Katyayani Sukhavasi, Rajeev Jain, Anamika Jain, Christer Betsholtz, Chiara Giannarelli, Jason C. Kovacic, Arno Ruusalepp, Josefin Skogsberg, Ke Hao, Eric E. Schadt, Johan L.M. Björkegren

**Affiliations:** 1 Integrated Cardio Metabolic Centre, Karolinska Institutet, Huddinge, Sweden; 2 Department of Cardiac Surgery, Tartu University Hospital, Tartu, Estonia; 3 Department of Pathophysiology, Institute of Biomedicine and Translational Medicine, University of Tartu, Tartu, Estonia; 4 Department of Immunology, Genetics and Pathology, Uppsala Universitet, Uppsala, Sweden; 5 Cardiovascular Research Center, Icahn School of Medicine at Mount Sinai, New York, NY, United States of America; 6 Institute of Genomics and Multiscale Biology, Department of Genetics and Genomic Sciences, Icahn School of Medicine at Mount Sinai, New York, NY, United States of America; 7 Clinical Gene Networks AB, Stockholm, Sweden; 8 Department of Medical Biochemistry and Biophysics, Karolinska Institutet, Solna, Sweden

**Keywords:** RNA editing, Gene expression, Quantitative trait loci, RNA-seq, Bioinformatics, Biostatistics

## Abstract

RNA editing modifies transcripts and may alter their regulation or function. In humans, the most common modification is adenosine to inosine (A-to-I). We examined the global characteristics of RNA editing in 4,301 human tissue samples. More than 1.6 million A-to-I edits were identified in 62% of all protein-coding transcripts. mRNA recoding was extremely rare; only 11 novel recoding sites were uncovered. Thirty single nucleotide polymorphisms from genome-wide association studies were associated with RNA editing; one that influences type 2 diabetes (rs2028299) was associated with editing in *ARPIN*. Twenty-five genes, including *LRP11* and *PLIN5,* had editing sites that were associated with plasma lipid levels. Our findings provide new insights into the genetic regulation of RNA editing and establish a rich catalogue for further exploration of this process.

## Introduction

Originally discovered in trypanosomes (Benne et al., 1986), RNA editing is a co-/post-transcriptional process that increases RNA diversity in eukaryotes. The most common modification in mammals is the deamination of adenosine to inosine (A-to-I), catalyzed by adenosine deaminases acting on RNA (ADAR) enzymes (Farajollahi & Maas, 2010). Inosine base pairs with cytosine and is interpreted as guanosine by the cellular machinery (Bass, 2002; Farajollahi & Maas, 2010). A-to-I editing occurs in many human tissues and acts on the majority of human pre-mRNAs (Park et al., 2012; Bahn et al., 2012; Bazak et al., 2014). Most RNA editing takes place in *Alu* repetitive elements. These short interspersed nuclear elements are ∼300 base pairs (bp) in length, specific to primates, and often embedded in introns and 3′ untranslated regions (UTRs). The abundance of *Alus* in the human genome—more than one million copies—makes it very likely to encounter inverted pairs of *Alus*, which can form stem-loop structures that are favored substrates of ADAR. Cytosine-to-uracil (C-to-U) editing is much less frequent than A-to-I editing (Blanc & Davidson, 2003) and is mediated by APOBEC enzymes. The medical implications of RNA editing may be extensive as it has been implicated in diseases ranging from cancer and neurological diseases (Hideyama et al., 2010; Hood & Emeson, 2011; Galeano et al., 2012; Slotkin & Nishikura, 2013) to atherogenesis (Stellos et al., 2016).

Detection of RNA-edited sites is relatively straightforward since the sequencing of an RNA-edited transcript (actually cDNA) will capture the edited base (Ramaswami & Li, 2016). A discrepancy in the alignment between the sequence and the genomic reference can be an RNA editing event, a single nucleotide polymorphism (SNP), or an artifact related to sequencing or data processing. Several computational methods have been devised to call RNA editing events from RNA-seq data (Ramaswami et al., 2013; Picardi et al., 2015; Zhang & Xiao, 2015; Kim, Hur & Kim, 2016; Ramaswami & Li, 2016; Tan et al., 2017).

Here, we analyzed transcriptome-wide RNA editing in one large cohort of patients with coronary artery disease—the Stockholm-Tartu Atherosclerosis Reverse Network Engineering Task (STARNET) (Franzén et al., 2016) study. We implemented a novel detection pipeline with several systematic filtering steps to deliver a refined set of RNA editing events. Our pipeline borrows ideas from published methods and it is designed for being executed on a computer cluster.

First, we focused on patterns of global RNA editing. To identify clinical features associated with RNA editing, we mapped RNA editing quantitative trait loci (edQTLs). Finally, to assess the disease relevance of RNA editing, we analyzed our edQTLs in the context of published genome-wide association studies (GWAS).

## Methods

### Statistics

R v.3.2.2 was used for all statistical analyses (R Core Team, 2015). If not otherwise specified, *P*-values were calculated with Welch’s *t*-test. All statistical tests were two-sided.

### Data sources

RNA-seq and genotype data (generated with the Illumina Infinium OmniExpressExome-8 chip) in this study originated from samples collected of up to 7 tissues (artery aorta, internal mammary artery, whole blood, subcutaneous fat, visceral fat, liver, and skeletal muscle) and 2 cell types (macrophages and foam cells) (Franzén et al., 2016) from 855 human subjects. Not all tissues/cell types were collected from all subjects. The rationale for collecting these tissues and their role in coronary artery disease have previously been described (Björkegren et al., 2015). Ethical permits, biopsy and experimental procedures have previously been described (Franzén et al., 2016) (Karolinska Institutet Dnr 154/7 and 188/M-12). The samples from aortic wall (*n* = 538) and internal mammary artery (*n* = 552) were exclusively prepared from rRNA-depleted libraries generated with the Illumina RiboZero protocol. The remaining samples were almost all poly(A)-enriched. Of the 4301 samples included in this study, 52% (2,267/4,301) were sequenced with a strand-specific protocol, and the remaining were sequenced with a non-strand-specific protocol. A complete summary of tissues, samples and protocols is shown in Tables S1 and S2. Editing calls are listed in Dataset S1.

Metabolite and protein data were generated by Olink AB (Sweden) and Nightingale (formerly Brainshake, Finland).

### Genome version, annotations and scripts

The human genome GRCh38 and GENCODE (Harrow et al., 2012) v.24 annotations were used throughout this study. RNA editing sites were mapped to genomic features with ANNOVAR (Wang, Li & Hakonarson, 2010) v.2015-03-22. miRBase (Kozomara & Griffiths-Jones, 2014) v.21 was used for miRNA annotations. Scripts used to process data can be retrieved from: https://github.com/oscar-franzen/rnaed.

### Computational scheme for detection of RNA editing sites

Sequencing reads were aligned with the human genome using STAR (Dobin et al., 2013) v.2.5.1b, with the maximum number of alignments set to 1, and the ratio of mismatches to sequence length set to 0.1. Only uniquely mapped reads were considered (mapping quality = 255). The strand specificity of sequencing was confirmed by counting reads mapped to sense and antisense transcripts with HTSeq (Anders, Pyl & Huber, 2014) v.0.6.0. For all samples, the predicted strandedness matched the recorded information from the sequencing facility. Only samples with more than 1,000,000 uniquely mapped reads in genes were analyzed. The first step in our pipeline was collapsing polymerase chain reaction (PCR) duplicates with the rmdup command in samtools v.1.2. This step limits the number of false positives from the library construction process (i.e., amplification of errors introduced by the reverse transcriptase). Alignments were then scanned for single nucleotide variants (SNVs) by parsing the output of the mpileup command in samtools. SNVs were removed if located within simple repeats, homopolymers ≥ 5 bp, within 5 bp of splice junctions. SNVs located on the mitochondrial genome, on unplaced contigs, and within the major histocompatibility complex were also removed. Intersections were called with bedtools (Quinlan & Hall, 2010) v.2.21.0. Furthermore, SNVs listed in any of the following databases were discarded: NCBI dbSNP b141, b146, and b147; Exome Aggregation Consortium variants v.0.3.1; NHLBI ESP6500 variants; Scripps’ Wellderly (Erikson et al., 2016); and COSMIC (Forbes et al., 2015) v.77. For the remaining SNVs, all sequencing reads overlapping the site were extracted and realigned with GSNAP (Wu & Nacu, 2010) v.2016-05-25, using settings that allow mismatches and splice junctions. For each sequencing read, the alignment coordinates (chromosome, strand, start and stop positions) from STAR and GSNAP were compared, and discordant alignments were removed. A concordant alignment was defined as >99% overlap of the start-stop interval on the same chromosome and strand. To call an RNA editing event, the base quality score had to be ≥20. To avoid errors introduced from random hexamer priming, the SNV had to be >6 bases from the 5′ start position of the sequencing read. SNVs indicating more than one type of variant per site were ignored. The final criteria required at least two non-identical sequencing reads to support the RNA editing event.

121.2 billion single-end reads were mapped to unique locations in the human genome (median  = 27,539,768 reads/sample). Collapsing PCR duplicates reduced the numbers to ∼42.9 billion aligned reads (median  = 9,527,872 reads/sample). In total, 22,124,713 RNA editing events were called, mapping to 2,631,392 unique sites. 83.3% (2,866,471/3,438,790) and 92.6% (17,304,283/18,685,923) of the identified events were A-to-G mismatches in strand-specific and non-strand-specific libraries, respectively. In the non-strand-specific data, these numbers also include the reverse complement of A-to-G (i.e., T-to-C).

### Quality of detected editing events

Sequencing errors are more likely to occur toward the end of reads. We did not find 5′ or 3′ positional biases in sequencing reads for canonical events (A-to-I and C-to-U; Fig. S13). Next, assuming that G-to-A events in strand-specific samples were sequencing or mapping errors, we estimated that the false discovery rate (FDR) was 3.1% (89,490/2,866,471). For non-strand-specific samples (assuming all G-to-C events were sequencing or mapping errors), the FDR was 0.83% (72,500/8,717,781).

Collected RNA samples from macrophages and foam cells are near biological replicates (foam cells are derived from macrophages by incubating them with acetylated LDL for 48 h). Therefore, we assessed the reproducibility of RNA editing in 235 macrophage—foam cell pairs. We first assessed the percentage of editing sites detected in both samples in each pair; the median across all pairs was 24%. It may therefore be plausible that many RNA editing sites are not strictly reproducible even in samples that are close to biological replicates. This may reflect the stochastic nature of RNA editing or variability in sequencing depth. We also determined whether the editing ratios were stable for the ∼24% of sites found in both samples of each pair. Across each of the 235 macrophage-foam cell pairs, the median Spearman correlation was 0.83 (Fig. S14). Thus, despite known technical and biological confounders, the reproducibility was relatively high for sites that can be detected in both samples.

### Gene expression

Gene expression was calculated as RPKM (Mortazavi et al., 2008) from reads counted with HTSeq (Anders, Pyl & Huber, 2014).

### Association-testing of editing ratios and clinical parameters

Association-testing was conducted with the linear model in MatrixEQTL (Shabalin, 2012) v.2.1.1. Clinical parameters were first rank-normal transformed with the function *rntransform* in the R package GenABEL (Aulchenko et al., 2007) with ties broken randomly. Two biological covariates (age and sex) and two technical covariates (sequencing batch and laboratory) were included in the statistical model. Only sites with non-zero A-to-G(I) editing ratios in more than 50 samples per tissue were considered. Significant associations were defined as those satisfying the following criteria: (i) adjusted *P* < 0.05 (10,000 permutations were executed for each clinical parameter-tissue combination); (ii) R-sq > 0.4; and (iii) no correlation between gene expression (of the overlapping gene) and the tested clinical parameter (nominal *P* > 0.05).

### RNA-editing quantitative trait loci (edQTLs)

Genotypic data were processed as described (Franzén et al., 2016); biallelic markers (MAF > 0.05) were converted to allele dosages (0, 1, and 2). edQTLs were generated separately for each tissue. Only A-to-G(I) sites found (i.e., editing ratio > 0) in more than 40 samples were included in the analysis (sites on sex chromosomes were excluded). Site-sample combinations with fewer than <5 reads supporting editing were removed. Editing ratios were then rank-normal transformed as described, and the null hypothesis of no association between allele dosages and editing ratios was tested with a linear regression model in MatrixEQTL (Shabalin, 2012). For each hypothesis, we required at least 20 subjects in at least 2 genotype-allele groups. Thus, the minimum number of individuals for any test was 40. All possible genotype-editing site combinations were tested regardless of the absolute genetic distance. We corrected for the following covariates: age, sex, laboratory, batch, and population stratification (i.e., cryptic relatedness of study subjects; represented as four genetic multi-dimensional scaling components). The FDR was controlled using a permutation procedure (Franzén et al., 2016): the RNA editing matrix was scrambled 1,000 times, i.e., breaking the link between sample identifiers and corresponding measurements. For each editing site, we generated 1,000 permutation *p*-values, which were used to adjust the real *p*-values. An adjusted *p*-value <0.20 was considered significant.

### Overlap with eQTLs

Normalized gene expression data from (Franzén et al., 2016) were used in a linear regression model. *P*-values were adjusted with the Benjamini–Hochberg procedure and adjusted *P* < 0.05 was considered significant. For each editing site, all overlapping genes were tested.

### GWAS SNPs

The NHGRI-EBI GWAS catalog (Welter et al., 2014) (r2018-01-01) and GWASdb (Li et al., 2016) v.2 were downloaded and merged into one database, keeping only SNPs with *P* < 1e − 08. GWAS SNPs that were also edSNPs were saved in a new list that was LD-pruned with PLINK (–indep-pairwise 400 kb 1 0.6) (Purcell et al., 2007).

## Results

### General characteristics of the RNA editome

**Figure 1:**
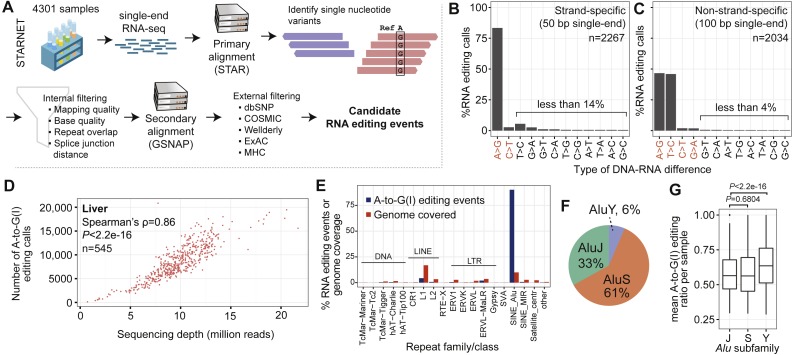
General characteristics of RNA editing events. (A) Simplified flowchart of the data analysis pipeline. See Fig. S1 for details. MHC, major histocompatibility complex. (B) Percentages of RNA editing types that were identified. The *x*-axis shows the editing type, and the *y*-axis shows the percentage of total editing calls. (The plot counts redundant sites, i.e., if the same RNA editing site is found in two samples, then the site is counted twice.) Editing events were identified in strand-specific libraries. Changes indicated in red (*x*-axis) are canonical events. The majority of events are consistent with A-to-I editing. (C) As (B) except that editing events were identified in non-strand-specific libraries. (D) Relationship between sequencing depth after collapsing of PCR duplicates (*x*-axis; million reads) and number of A-to-G(I) editing calls (*y*-axis). Each dot represents a liver sample from one subject. Only the non-strand-specific samples are shown. (E) Percentage of A-to-G(I) events in various genomic repeats. The repeat family/class is shown on the *x*-axis and identified events/genomic coverage in percent is on the *y*-axis. *Alu* is the only repeat class that shows enrichment compared with the total genomic coverage. (F) Pie chart of A-to-G(I) events in* Alu* subfamilies (S, Y, and J). (G) The box plot shows sample mean editing ratios of A-to-G(I) events. One outlier was removed from the Y subfamily. Editing events in *Alu* Y have significantly higher editing ratios compared with those in *Alu* J and S (Mann–Whitney *U* test; *P* < 2.2e − 16).

To characterize the human RNA “editome”, we analyzed RNA-seq data from the STARNET (Franzén et al., 2016) study, in which samples were obtained from up to 9 tissues (atherosclerotic aortic wall, *n* = 538; non-atherosclerotic arterial wall (internal mammary artery), *n* = 552; liver, *n* = 545; skeletal muscle, *n* = 533; visceral abdominal fat, *n* = 533; subcutaneous fat, *n* = 532; whole blood, *n* = 559; macrophages, *n* = 256; and foam cells, *n* = 235). In addition, 18 coronary artery samples were included. Sequencing was done by using 50-bp single-end reads (these libraries were strand-specific; *n* = 2, 267) and 100-bp single-end reads (these libraries were non-strand-specific; *n* = 2,034). Differences in read-length and strand specificity affected the sensitivity to detect RNA editing, and most analyses were therefore done separately on these two sets of samples. See Table S1 for a summary of analyzed samples. We applied a rigorous computational pipeline that includes internal and external filters to identify RNA editing in a total of 4,301 RNA-seq samples (Fig. 1A; Fig. S1). The number of samples per tissue ranged from 235 in foam cells to 559 in whole blood. For each putative editing site, we calculated the RNA editing ratio, defined as the count of edited bases divided by the total number of bases covering the site. The RNA editing ratio takes values within the semi-closed interval (0,1].

Most of the identified events were A-to-G mismatches (Figs. 1B and 1C; Table 1; Tables S2 and S3), consistent with A-to-I editing; henceforth we refer to these events as A-to-G(I) editing (including the reverse complement when the strand information was not preserved). We identified 1,484,357 and 450,002 A-to-G(I) events in non-strand-specific and strand-specific libraries, respectively; the number of non-redundant A-to-G(I) editing events in non-strand-specific and strand-specific libraries was 1,688,815. More A-to-G(I) events were detected in non-strand-specific libraries, owing to deeper sequencing and longer read length. Liver had the highest number of A-to-G(I) events, possibly reflecting higher transcriptional complexity. In total, 53.9% (910,943/1,688,815) of identified A-to-G(I) events had previously been reported in REDIportal (Picardi et al., 2017) or DARNED (Kiran & Baranov, 2010) (Fig. S2). Aortic wall had the highest number of novel A-to-G(I) sites (*n* = 117, 493). A-to-G(I) editing was detected in transcripts from 25,951 genes, of which 62.1% (16,131/25,951) were protein-coding. We intersected RNA editing events with genome annotations (Fig. S3), showing that 56.0% of A-to-G(I) events were in introns, 18.3% in intergenic regions, and 9.4% in 3′ UTRs; 67.7% of the A-to-G(I) editing sites were tissue-specific when comparing exact chromosome-positions without considering differences in gene expression (Fig. S4). To exclude bias from differences in gene expression we repeated the analysis and only examined genes being robustly expressed in 7 tissues (defined as median reads per kilobase of transcript per million mapped reads [RPKM] >10 across all samples; 2,639 genes), the result showed that most events were tissue-specific (Fig. S4).

**Table 1:** Summary of data and RNA editing events.

Library type	Seq. read length (bp)	No. samples	Seq. depth (×10^9^)^a^	Total no. RNA editing events	No. A-to-G(I) events	No. C-to-T(U) events
Strand-specific	50	2,267	20.6	714,372	450,002	40,116
Non-strand-specific	100	2,034	22.3	2,217,412	1,484,357^b^	375,935^c^

**Notes.**

a Total read count after collapsing of PCR duplicates (billion single-end sequencing reads).

b Includes T-to-C calls.

c Includes G-to-A calls.

In strand-specific samples, the second most common change was noncanonical T-to-C editing (16.2%, 116,318/714,372). Interestingly, in strand-specific libraries the number of A-to-G(I) events correlated with the number of T-to-C events (Spearman’s *ρ* = 0.67, *P* < 2.2e−16). T-to-C events have been reported in studies utilizing high-throughput sequencing data but it is unknown whether T-to-C events are authentic or artifacts (Bahn et al., 2012).

The relationship between sequencing depth and number of editing events was nearly linear (Fig. 1D and Fig. S5; liver samples: Spearman’s *ρ* = 0.86, *P* < 2.2e−16), suggesting that detection of RNA editing per sample was not saturated or that the mechanisms mediating RNA editing are nonspecific. If RNA editing is nonspecific, it can be assumed that deeper sequencing does not saturate detection. Analysis of the cumulative number of unique A-to-G(I) sites as a function of added samples did not reveal a clear tendency toward saturation (Fig. S6A). It is also possible that saturation cannot be detected because most editing takes place in introns, which are much less captured with RNA-seq. However, analysis of the number of genes in which RNA editing occurred did reveal a trend toward saturation (Fig. S6B).

### Differential editing in *Alu* subfamilies

In the present version of the human genome assembly (GRCh38) there are 1,186,920 *Alus* covering 10.2% (315,412,481 bp/3,088,269,832 bp). *Alu* was the sole element significantly enriched in editing events (Fig. 1E; Figs. S7–S9). Overall, 70.3% (1,187,358/1,688,815) of all A-to-G(I) events were in *Alu*s. Of the *Alus* in the human genome, 20.3% (241,226/1,186,920) had at least one A-to-G(I) editing event. Despite covering 20.7% (641,953,033 bp/3,088,269,832 bp) of the genome (Lander et al., 2001), LINE elements contained only 4.0% (67,810/1,688,815) of the A-to-G(I) events. The mean editing ratio per sample was slightly lower in non-*Alu* A-to-G(I) compared to editing sites in *Alus* (mean_non−Alu_ = 0.54, mean_Alu_ = 0.57, *P* = 3.5e−15), likely reflecting the strong preference of ADAR for double-stranded folds. When *Alu* elements were grouped by subfamilies (Y, J, and S) according to their established phylogeny (Bennett et al., 2008), it showed that 42.5% (718,653/1,688,815) of A-to-G(I) editing events were located within the *Alu*S subfamily, followed by *Alu*J (23.2%) and *Alu*Y (4.5%), see Fig. 1F. The mean editing ratio per sample was highest in *Alu*Y (mean_*Alu*S_ = 0.56, mean_*Alu*J_ = 0.56, mean_*Alu*Y_ = 0.64, *P* < 2.2e−16; Fig. 1G). Mean editing levels were also significantly higher within nongenic *Alus* compared to genic *Alus* (mean_genic*Alus*_ = 0.56, mean_nongenic*Alus*_ = 0.66, *P* < 2.2e−16), perhaps because purifying selection lowered editing levels in mRNAs.

### *ADAR1* is ubiquitously expressed and *ADAR2* displays a vascular-specific signature

**Figure 2:**
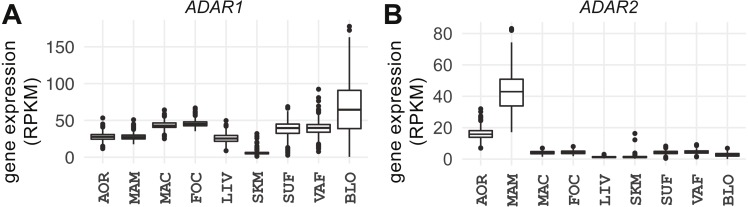
Expression of *ADAR1* and *ADAR2* across studied tissues. Box plots show gene expression of (A) *ADAR1* and (B) *ADAR2* in RPKM (*y*-axis). AOR, aorta; MAM, internal mammary artery; MAC, macrophage; FOC, foam cell; LIV, liver; SKM, skeletal muscle; SUF, subcutaneous fat; VAF, visceral fat; BLO, whole blood.

ADAR1 and ADAR2 catalyze A-to-I editing (Macbeth et al., 2005; Nishikura, 2016). ADAR3 is catalytically inactive and may have regulatory properties (Chen et al., 2000). The expression of *ADAR1*, *ADAR2*, and *ADAR3* was compared across the tissues/cell types (Fig. 2; Fig. S10). *ADAR1* was uniformly expressed in all tissues (median = 31 RPKM, median absolute deviation [MAD] = 14); the lowest levels observed were in skeletal muscle (median = 5 RPKM, MAD =1 ), see Fig. 2A. The latter was also observed in GTEx (Melé et al., 2015). Expression of *ADAR1* correlated with A-to-G(I) editing events in every tissue except foam cells (*q*-value < 0.05), further corroborating the authenticity of called RNA editing events. Whole blood showed the strongest correlation between A-to-G(I) editing events and *ADAR1* expression (strand-specific samples, Spearman’s *ρ* = 0.85, *P* < 2.2e−16; Fig. S11). Although sex-specific differences in *ADAR1* expression have been reported in tumor samples (Paz-Yaacov et al., 2015), we found only modest sex-specific differences in foam cells (*P* = 0.015), liver (*P* = 0.049), and subcutaneous fat (*P* = 0.017; Fig. S12).

*ADAR2* expression was low in all tissues (Fig. 2B; median = 3 RPKM, MAD = 2) except the three vascular tissues (atherosclerotic aortic wall, internal mammary artery, and coronary artery; median = 23 RPKM, MAD = 14); expression was highest in internal mammary artery (median = 42 RPKM, MAD = 12). *ADAR2* expression correlated with the number of A-to-G(I) events only in atherosclerotic aortic wall (Spearman’s *ρ* = 0.18, *P* = 2.6e−05) and was stronger in females (females: *ρ* = 0.19, *P* = 0.012; males: *ρ* = 0.17, *P* = 5.2e−04). However, there were no sex-specific differences in editing in any tissues (*P* > 0.05). *ADAR3* was essentially below the detection level in all tissues (median = 0.01 RPKM, MAD = 0.02).

### Eleven novel recoding sites and one loss of stop codon

RNA editing provides a mechanism for cells to generate additional protein diversity (Nishikura, 2010). However, novel protein recoding events—those that cause amino acid changes—are rare. We systematically searched for A-to-G(I) editing events causing recoding. We found that 3.9% (67,429/1,688,815) of A-to-G(I) events were within known protein-coding exons (variants arising from antisense transcription were excluded), and only 3.0% (50,883/1,688,815) were non-synonymous substitutions. The latter appeared in a total of 9,739 protein-coding genes. The median number of non-synonymous substitutions per sample was 18 (MAD = 19).

Editing ratios had a long-tailed skewed distribution (median = 0.14), suggesting that these variants are present in a small number of transcripts. Most of the non-synonymous sites (90.8%, 46,245/50,883) were found in single samples. Thus, although variants are pervasive on a global scale, many reflect background noise.

To focus on likely functional sites, we looked for conserved non-synonymous substitutions, that were present in >20 tissue samples and had a median editing ratio >0.1 in any tissue. Using these criteria, we found 65 conserved editing sites in 44 genes (Table S4). Six sites were predicted to be deleterious according to PROVEAN (Choi et al., 2012) and SIFT (Ng & Henikoff, 2001) (chr4:57,110,146, chr6:44,152,612, chr9:33,271,197, chr16:5,044,722, chr16:57,683,958 and chr17:1,534,142). As expected, conserved editing sites had higher editing ratios (median_conserved_ = 0.30, median_notconserved_ = 0.05, *P* < 2.2e−16, Mann–Whitney *U* test). Moreover, 80% (52/65) of these sites had been reported in REDIportal (Picardi et al., 2017), and 35% (23/65) had been reported in the literature (Table S4). The classical example of recoding in *GRIA2* (glutamate ionotropic receptor AMPA type subunit 2) was found at the known site (chr4:157,336,723; p.Q607R) (Wright & Vissel, 2012) in aortic wall and internal mammary artery. The median editing ratio in each tissue was 1, indicating that all transcripts carried the modified base.

Eleven recoding sites were novel (Table 2). Seven had low editing ratios (<0.5; median = 0.24), which in part may explain why they were not detected previously. This finding highlights the power of large RNA-seq datasets such as STARNET.

**Table 2:** Novel A-to-G(I) mRNA-recoding sites.

Site^a^	Gene	Amino acid change	Tissue(s)^b^	Median editing ratio^c^	N^d^
4:57,110,146	*IGFBP7*	E69G	AOR, MAM	0.11	197
7:38,262,191	*TARP*	N58S	BLO	0.40	37
8:144,247,668	*MROH1*	S1037G, S1028G	LIV, VAF	0.47	94
9:33,271,197	*CHMP5*	K121E	BLO, SUF	0.07	853
10:45,789,442	*FAM21C*	K1158R, K1124R, K1199R	BLO, LIV, SUF, SKM, VAF	0.50	216
12:57,625,434	*SLC26A10*	R500G	MAM	0.29	74
16:30,188,879	*CORO1A*	E434G	VAF	0.17	22
17:1,534,142	*PITPNA*	D242G	LIV	0.08	55
19:7,520,407	*ZNF358*	S389G	MAM	0.09	95
19:54,221,229	*LILRB3*	Q270R	FOC, MAC	0.21	251
19:54,221,256	*LILRB3*	E261G	FOC, MAC	0.50	223

**Notes.**

a Genomic coordinate of the site (GRCh38). Given as chromosome:position.

b Tissue abbreviations: aortic wall (AOR), internal mammary artery (MAM), whole blood (BLO), liver (LIV), visceral fat (VAF), subcutaneous fat (SUF), skeletal muscle (SKM), foam cells (FOC), and macrophages (MAC).

c Across all samples.

d Number of samples in which editing was found. This number may include samples whose editing signal was lower than required to meet the criteria for detection.

Two recoding sites (one previously reported Zhang et al., 2014a and one novel) resided in *IGFBP7* (insulin-like growth factor-binding protein 7). The first site, chr4:57,110,062 changes a codon encoding lysine to arginine (amino acid 97); it was found specific to the internal mammary artery and had been reported in brain (Zhang et al., 2014a). The second, chr4:57,110,146, changes a codon encoding glutamic acid to glycine (amino acid 69); it was found in both atherosclerotic aortic wall and the internal mammary artery. Both recoding sites were in the IGF-binding domain of IGFBP7, possibly highlighting a novel regulatory mechanism.

Two recoding sites (chr2:219,483,602 in *SPEG* and chr3:179,375,226 in *MFN1*) were previously reported only in mouse (Danecek et al., 2012), suggesting these mRNA-recoding sites are of ancient origin since they were present in the common ancestor of extant human and mouse lineages (diverged ∼65 to 110 million years ago (Emes et al., 2003)).

Next, we investigated A-to-I recoding events resulting in the loss of stop codons (the opposite, the gaining of stop codons, is not biologically possible by A-to-I editing). Using the same criteria described above, we found one recoding site, at chr10:101,017,585 in *PDZD7* (PDZ domain containing 7). It was specific to visceral abdominal fat and modifies the stop codon UAG to UGG (encodes tryptophan), extending PDZD7 by 18 amino acids (WSQIPGLKLSTRLSLPKC). This recoding site was recently reported in human brain tissue (Hwang et al., 2016). To our knowledge this is the first and perhaps only recoding site in humans that causes protein extension. We checked if the change was associated with measured traits (e.g., LDL-C) and we did not find any association.

### pri-mir-27b and mir-605 editing in aortic wall and internal mammary artery are associated with increased plasma levels of high-density lipoprotein

In preparing the sequencing libraries, we used the Ribo-Zero protocol to eliminate ribosomal RNA, which enabled us to investigate small non-coding RNAs in 996 RNA-seq samples from atherosclerotic aortic wall and internal mammary artery. We identified A-to-G(I) editing sites in 102 unique microRNAs (miRNAs; annotations span the entire predicted stem-loop region) and 73 small nucleolar RNAs (snoRNAs). Stringent editing criteria (recurrence in > 50 samples) identified 18 editing sites in 10 miRNAs (mir-24-2, mir-27a, mir-27b, mir-605, mir-641, mir-1254, mir-1285-1, mir-1304, mir-3126, and mir-3138; Table S5) and 2 snoRNAs (U88 and SNORD17). RNA editing sites in mir-24-2, mir-605, mir-3126, and U88 had not previously been reported in REDIportal (Picardi et al., 2017) or DARNED (Kiran & Baranov, 2010). Six editing sites occurred in mature miRNAs: three in mir-605-3p and one each in mir-3138, mir-605-5p, and mir-24-2-3p. Two editing sites were associated with higher HDL cholesterol levels: chr9:95,085,457, which was located outside the mature miRNA (pri-mir-27b; *P* = 2.2e−05; FDR = 8.5%) and chr10:51,299,636 (mir-605-3p; *P* = 1.6e−06; FDR = 0.65%).

### RNA editing in 3′ UTRs of several genes is associated with plasma lipid levels

Next, we looked for associations between 18,458 RNA editing sites and 208 clinical parameters (Franzén et al., 2016). Thirty-nine associations were found significant (FDR < 5%; 10,000 permutations) and involved 25 genes in five tissues (Table S6). 52% (13/25) of these were located in 3′ UTRs, implying they could affect transcript regulation. Of the remaining sites, eight were in introns, two were in known regulatory elements, one was exonic, and three were intergenic. A subset of all significant associations is listed in Table 3.

**Table 3:** A-to-G(I) RNA editing sites associated with clinical parameters.

Site^a^	Tissue^b^	Trait	*P*-value	FDR^c^	Gene	Region
1:204,556,653	LIV	HDL	2.7e−06	0.04	*MDM4*	3′ UTR
2:37,100,523	BLO	LDL	5.1e−06	0.03	*EIF2AK2*	3′ UTR
6:149,822,432	SUF	LDL	7.0e−06	0.01	*LRP11*	Intron
10:50,804,961	LIV	LDL/HDL	5.7e−07	0.01	*A1CF*	3′ UTR
12:7,097,577	LIV	LDL/HDL	5.9e−07	0.01	*C1RL*	Intron
12:68,843,961	LIV	18:2, linoleic acid	8.2e−08	0.003	*MDM2*	3′ UTR
19:4,526,500	LIV	HDL	3.1e−07	0.005	*PLIN5*	Intron
19:6,683,589	LIV	HDL	2.7e−06	0.04	*C3*	Intron, RE^d^
19:11,449,682	LIV	VLDL	1.4e−06	0.02	*PRKCSH*	Intron

**Notes.**

a Genomic coordinate of the site (GRCh38). Given as chromosome:position.

b Tissue abbreviations: aortic wall (AOR), internal mammary artery (MAM), whole blood (BLO), liver (LIV), visceral fat (VAF), subcutaneous fat (SUF), skeletal muscle (SKM), foam cells (FOC), and macrophages (MAC).

c False discovery rate (permutation *P*-value). Based on 10,000 permutations.

d Regulatory element (RE).

Although none of the 25 genes had been directly associated with coronary artery disease, 22 of 25 sites were associated with plasma lipoprotein levels. Two of the affected genes may have roles in lipid metabolism: *LRP11* (low density lipoprotein receptor-related protein 11) and *PLIN5* (lipid storage droplet protein 5). In subcutaneous fat, the *LRP11* editing site (chr6:149,822,432), located in the most 3′ intron of the longer isoform, was associated with plasma low density lipoprotein (LDL) levels. RNA editing of two genes known for their roles in innate immune response—*C3* (complement component 3) and *C1RL* (complement C1r subcomponent like)—occurred in liver and were both associated with plasma HDL levels.

### QTL mapping of RNA editing identifies novel candidate disease genes

**Figure 3:**
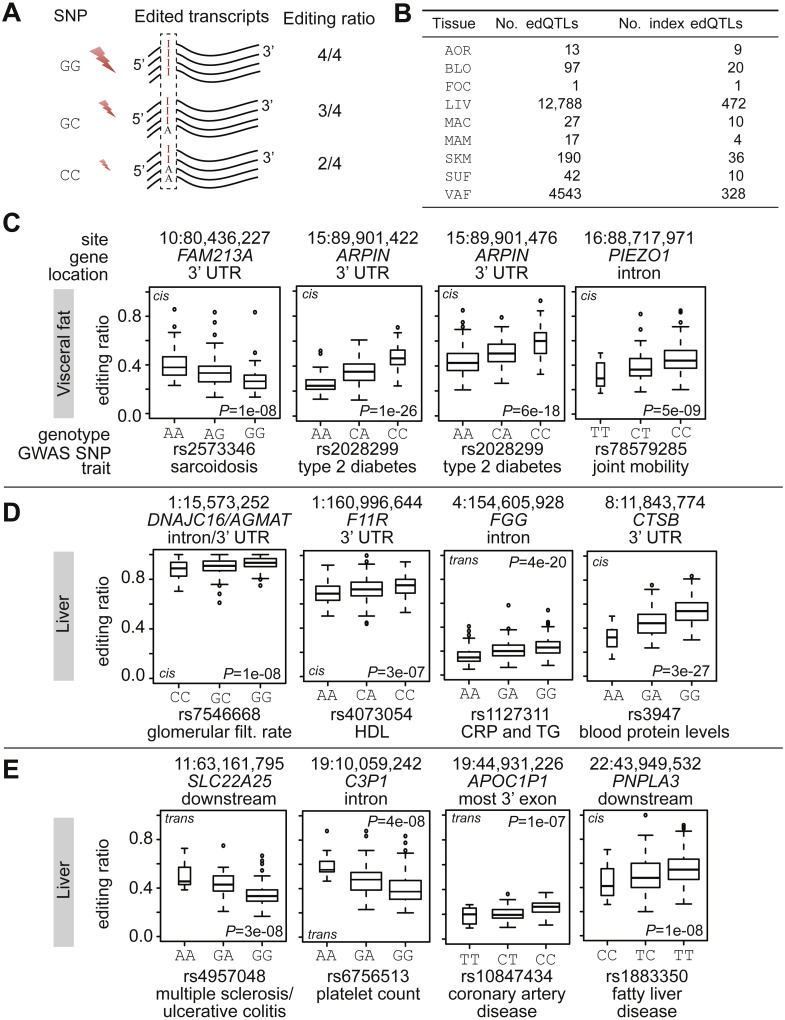
RNA editing quantitative trait loci. (A) Schematic illustration of an RNA editing QTL (edQTL). The genomic marker (e.g., a SNP) is associated with RNA editing levels at a specific site in a transcript. (B) Summary of number of identified (index) edQTLs per tissue. (C–E) Box plots of edQTLs where regulatory SNPs coincide with lead SNPs from GWAS. *P*-values refer to non-rank-normal transformed data. The genomic coordinate of the RNA editing site and the overlapping gene are shown on top of each plot. The GWAS SNP is shown at the bottom together with its reported trait. The width of the box is proportional to the number of observations it contains. Rings are outlier samples. The molecular distance is indicated on the top of each subplot: (*cis*) intra-chromosome association; and (*trans*) inter-chromosome association. GWAS *P*-values: rs2573346 (sarcoidosis), *P* = 9e−16; rs2028299 (type 2 diabetes), *P* = 1e−11; rs78579285 (joint mobility), *P* = 6e−12; rs7546668 (glomerular filtration rate), *P* = 1e−09; rs4073054 (HDL), *P* = 5e−11; rs1127311 (C-reactive protein or triglycerides), *P* = 6e−09; rs3947 (blood protein levels), *P* = 2e−27; rs4957048 (multiple sclerosis/ulcerative colitis), *P* = 1e−09; rs6756513 (platelet count), *P* = 7e−10; rs10847434 (coronary artery disease), *P* = 6e−15; and rs1883350 (fatty liver disease), *P* = 4e−10.

To uncover possible genetic regulation of RNA editing (Kurmangaliyev, Ali & Nuzhdin, 2015), we looked for RNA editing quantitative trait loci (edQTLs; Fig. 3A). Using data from the 1000 Genome Project (Durbin et al., 2010), imputation resulted in 6,246,842 common variants with minor allele frequency >5%. Next, we identified 17,718 edQTLs at FDR <20% (i.e., associations between allele dosages and RNA editing ratios; Fig. 3B) by performing ∼2.6 billion hypothesis tests (comprising 11,280 unique editing sites). We examined whether identified edQTLs were also expression QTLs (eQTLs). 19.5% (3,465/17,718) of RNA editing sites in edQTLs were eQTLs with respect to the overlapping gene. Across all tissues, we found 890 index edQTLs—the most significant association for each RNA editing site (Dataset S2). A total of 77% (690/890) of the index edQTLs were *trans* (the regulatory SNP and the editing site are located on different chromosomes).

Most of the editing sites with edQTLs were enriched in *Alu* elements (87%, 783/890); the *Alu*-subtype distribution was the same as shown in Fig. 1F. A total of 95% (853/890) of the edQTLs have previously reported editing sites (Kiran & Baranov, 2010; Picardi et al., 2017); 47% (425/890) were in 3′ UTRs, 25% (230/890) were in introns, and 12% (113/890) were in intergenic regions. RNA editing sites of edQTLs overlapped 417 genes (Dataset S2).

To assess a possible mechanistic role of edSNPs in disease, we intersected edSNPs with lead SNPs from GWAS. 90 edSNPs coincided with GWAS SNPs, encompassing 59 traits. LD-pruning collapsed the 90 SNPs to 30 non-linked SNPs. rs2573346 is associated with sarcoidosis (Hofmann et al., 2008) and had an edQTL in the 3′ UTR of *FAM213A* in visceral fat (Fig. 3C). rs2028299 is associated with type 2 diabetes (Kooner et al., 2011) and had two edQTLs in visceral fat (both located in the 3′ UTR of *ARPIN*). rs2028299 is associated with expression of *ARPIN* in fat tissue (*P* < 1e−10) (Kooner et al., 2011). A negative correlation was found between *ARPIN* expression in visceral fat and plasma levels of VLDL (*r* =  − 0.2; *P* < 1e−06). rs78579285 has been associated with joint mobility (Beighton score) (Pickrell et al., 2016) and we detected an edQTL in visceral fat within the intron of *PIEZO1* (encodes a mechanosensitive ion-channel component). rs7546668 is associated with kidney function (glomerular filtration rate) (Gorski et al., 2017) and it formed an edQTL with an editing site detected in liver (Fig. 3D). The editing site overlapped *DNAJC16* (encodes DnaJ Heat Shock Protein Family (Hsp40) Member C16) and *AGMAT* (encodes Agmatinase). rs4073054 is associated with HDL (Chasman et al., 2009) and had an edQTL with an RNA editing site in the 3′ UTR of *F11R* in liver. Expression of *F11R* was associated with plasma levels of VLDL (*r* =  − 0.2, *P* < 1e−08) and HDL (*r* =  − 0.2, *P* < 1e−07). rs1127311 is a pleiotropic locus and it is associated with C-reactive protein and triglyceride levels (Ligthart, Vaez & Hsu, 2016). Interestingly, rs1127311 is situated in the 3′ UTR of *ADAR*, and it forms an edQTL with an editing site in the second to the last intron of *FGG*. rs3947, situated in the 3′ UTR of the protease-encoding gene *CTSB*, is associated with blood protein levels (Suhre et al., 2017). rs3947 forms an edQTL with an editing site located in the 3′ UTR of *CTSB*. rs3947 is in strong LD with rs2740594 (R-sq = 0.86), which is associated with Parkinson’s disease (Chang et al., 2017). rs4957048 is associated with multiple sclerosis/ulcerative colitis (De Lange et al., 2017), and it was associated with an editing site located downstream of *SLC22A25* (Fig. 3E). rs6756513 is associated with platelet count (Astle et al., 2016) and it had an edQTL in an intron of *C3P1*. rs10847434 is associated with coronary artery disease (Lee et al., 2013) and had an edQTL with an editing site in the most 3′ exon of *APOC1P1* in liver. *APOC1P1* encodes a long non-coding RNA without any known function or role. However, its locus (Jeemon et al., 2011) has been extensively linked to coronary artery disease (neighboring genes are *APOE*, *APOC1*, and *TOMM40*). In liver, there was a negative correlation between expression of *APOC1P1* and *APOE* (*r* =  − 0.3; *P* = 7e−13) as well as *APOC1P1* and *APOC1* (*r* =  − 0.22; *P* = 1e−07). The negative correlation may indicate a regulatory function of *APOC1P1*. rs1883350 is associated with fatty liver disease (Feitosa et al., 2013) and we identified one liver edQTL slightly downstream of *PNPLA3* (Patatin-like phospholipase domain-containing protein 3). *PNPLA3* is associated with fatty liver disease (Speliotes et al., 2010) and encodes a protein known to be involved in liver fat storage. Finally, rs4739066 is associated with myocardial infarction (Wakil et al., 2016) and we found this SNP to have two edQTLs with editing sites in the 3′ UTR of the *α*-tocopherol transfer protein gene (*TTPA*). The editing sites are located 64 bp apart and are intriguing since they have opposite effect sizes. *TTPA* is associated with the severity of atherosclerotic lesions in the proximal aorta (Terasawa et al., 2000).

## Discussion

We analyzed RNA editing by revisiting RNA-seq data from the STARNET study (Franzén et al., 2016). The study aimed to characterize the RNA editing landscape of human tissues and link RNA editing events with phenotypic traits. We analyzed several thousand RNA-seq samples using a novel detection pipeline for RNA editing. Our analysis covered 855 subjects, seven human tissues and two cell types. We identified ∼1.6 million RNA editing events, representing a strong A-to-I editing signal. The identified events constituted novel editing sites and previously reported sites (Picardi et al., 2017). Many novel events originated from intergenic regions and non-coding RNAs, indicating that many transcripts of the human genome are still uncharacterized. The A-to-I editing levels ranged from almost undetectable to 100%, suggesting that unidentified factors regulate the strength of editing at individual sites. As expected, the second type of canonical editing, C-to-U, was much less common (∼3.5% of identified events). Similar to previous studies (Park et al., 2012; Ramaswami et al., 2012; Ramaswami et al., 2013) we identified a large number of noncanonical edits, which may be true editing events, ultra-rare genetic variants or artifacts from sequencing and data processing. Noncanonical U-to-C was the second most common event (Fig. 1B) as shown by analysis of strand-specific data, confirming a previous report (Bahn et al., 2012). This finding may point to a novel editing mechanism in humans. The existence of putative U-to-C editing remains uncertain, and informatics alone is unlikely to settle this question.

We also found RNA editing to be strongly enriched in *Alu* elements, as previously reported (Athanasiadis, Rich & Maas, 2004; Kim et al., 2004; Ramaswami et al., 2012). Within and outside of *Alu* elements the pattern of editing was stochastic, reflecting the lack of specificity of the RNA editing machinery. The relationship between the number of events and sequencing depth also suggested that editing is unspecific, since there was no tendency to saturation. Most of the events were tissue-specific, likely reflecting differences in gene expression between tissues. Functional RNA editing sites have been reported in several studies, e.g., (Sommer et al., 1991). We took advantage of our multi-sample cohort to identify functional sites. Remarkably, across 1.6 million RNA editing sites, we discovered only 11 new sites that alter the amino acid sequence. Such recoding sites were inferred from their occurrence in multiple subjects. Sequence analysis revealed that most of them were neutral, indicating the role of purifying selection in removing harmful sites. Notably, *GRIA2* mRNAs were edited in aortic wall and internal mammary artery. *GRIA2* editing converts a codon for glutamine to arginine, which renders ion-channels impermeable to calcium ions. *GRIA2* is ubiquitously expressed in brain tissues as well as in the aortic wall, fallopian tubes, pituitary gland, and uterus (Melé et al., 2015). The role of these receptors outside the central nervous system is intriguing yet unknown.

We found that 39 RNA editing sites were associated with traits, raising the possibility of phenotypic consequences. In most cases editing was found in the 3′ UTR or an intron. The former is a target for transcript regulation, and may suggest disruption of binding sites of miRNAs or RNA-binding proteins. Several sites were found in introns, without obvious evidence for perturbed regulatory elements. While we are not able to rule out that some of these associations may have arisen due to unmeasured confounding factors, we believe further investigation is warranted.

Several A-to-I editing events were in important miRNA families, possibly as a mechanism to regulate miRNA function through modification of binding specificity. We found an association between plasma HDL levels and editing of pri-mir-27b/mir-605. mir-27b has been linked to progression of atherosclerosis (Li et al., 2011a; Zhang et al., 2014b), and mir-605 is implicated in stroke (Yuan et al., 2016) and hypertension (Li et al., 2011b). These data highlight the need to further integrate A-to-I editing data with respect to the short non-coding transcriptome. More specialized sequencing assays will be needed to capture the full extent of A-to-I editing in miRNAs and other small non-coding RNAs.

Finally, to examine the genetic regulation of RNA editing, we generated and examined edQTLs. edQTLs link RNA editing to genetic loci and have been described in *Drosophila melanogaster* (Kurmangaliyev, Ali & Nuzhdin, 2015; Ramaswami et al., 2015) and humans (Park et al., 2017). Intersecting regulatory SNPs with known disease-associated variants revealed putative causal links for several GWAS SNPs. We found 30 disease-associated regulatory SNPs, several of which have been linked to cardiometabolic traits. For example, rs2028299, previously associated with type 2 diabetes, was an edQTL in the transcript of *ARPIN*, suggesting dysregulation of RNA editing as the cause of increased disease risk.

## Conclusion

Our data indicate that RNA editing events are extremely common, but rarely of biological significance. Nevertheless, in certain instances such as among single nucleotide polymorphisms from genome-wide association studies, and for specific genes in disease-relevant tissues, RNA editing appears likely to play an important and potentially causal role in disease pathobiology. These findings highlight a novel mechanism of genetic contribution to cardiometabolic disease, and suggest that further investigation of this process is warranted.

##  Supplemental Information

10.7717/peerj.4466/supp-1 Supplemental Information 1 Supplementary figures and tables Figures S1–S14, Tables S1–S5.

10.7717/peerj.4466/supp-2 Supplemental Information 2 Associations between A-to-G(I) editing and clinical parameters

10.7717/peerj.4466/supp-3 Dataset S1 Supplementary material, RNA editing in metabolic and vascular human tissues List of discovered RNA editing events Supplementary material, RNA editing in metabolic and vascular human tissues The table is tab separated. The columns correspond to: (1) three letter tissue abbreviation; (2) library type (N = non-strand- specific, Y = strand-specific); (3) chromosome; (4) position; (5) DNA base; (6) RNA base; and (7) number of samples in this tissue-library combination where this event was detected. Coordinates are in GRCh38.

10.7717/peerj.4466/supp-4 Dataset S2 Supplementary material, RNA editing in metabolic and vascular human tissues List of identified RNA editing QTLs Supplementary material, RNA editing in metabolic and vascular human tissues The table is tab separated. The columns correspond to: (1) three letter tissue abbreviation; (2) regulatory SNP; (3) the encoded (effect) allele of the regulatory SNP; (4) genomic coordinate of the RNA editing site (GRCh38); (5) molecular interaction type (cis = the regulatory SNP and the editing site are on same chromosome, trans = the regulatory SNP and the editing site are on different chromosomes); (6) beta coefficient; (7) *p*-value of the association between RNA editing and the regulatory SNP; (8) adjusted (permutation-based) *p*-value; (9) the gene overlapping the RNA editing site; (10) same as previous, but giving the gene symbol instead; (11) gene biotype according to GENCODE; (12) the repeat type, if any, overlapping the RNA editing site; (13) the trait, if this regulatory SNP is a reported GWAS lead SNP; (14) gene region; (15) if the gene has previously reported as involved in cardiometabolic traits; (16) if the RNA editing site has been reported in DARNED; (17) if the RNA editing site has been reported in REDIportal; and (18) if the overlapping gene has an eQTL. Abbreviations: (Y)es, (N)o. This table is given as an external file.

## Additional Information and Declarations

### Data Availability

The following information was supplied regarding data availability: Code is available in GitHub: https://github.com/oscar-franzen/rnaed.
