# Supplementary material for: Global analysis of A-to-I RNA editing reveals association with common disease variants
Source: PeerJ. 2018 Mar 6;6:e4466. doi: 10.7717/peerj.4466 (PMC5844249; doi:10.7717/peerj.4466)
Supplement: Supplemental Information 1 — Figures S1–S14, Tables S1–S5. [file peerj-06-4466-s001.pdf]

Supplementary material

Global analysis of A-to-I RNA editing reveals association with common disease variants

Oscar Franzén, Raili Ermel, Katyayani Sukhavasi, Rajeev Jain, Anamika Jain, Christer Betsholtz, Chiara Giannarelli, Jason C. Kovacic, Arno Ruusalepp, Josefin Skogsberg, Ke Hao, Eric E. Schadt, Johan L.M. Björkegren

List of supplementary figures

Flowchart of the pipeline used to call RNA editing events . . . 3
Intersection of A-to-G(I) RNA editing events with public databases. . . . 4
Genomic annotation of single nucleotide variants. . . . 5
Percent A-to-G(I) events found in different number of tissues. . . . 6
Scatter plots showing the relationship between sequencing depth and number of called RNA editing events. 7
Cumulative number of detected A-to-G(I) editing events and genes. . . . 8
A-to-G(I) events falling in different repeat classes. . . . 9
A-to-G(I) events falling in *Alu* subtypes. . . . 10
C-to-T(U) events falling in various repeats. . . . 11
Comparison of *ADAR* expression across tissues. . . . 12
Scatterplot of *ADAR* expression in whole blood versus number of identified A-to-G(I) events. . . . 13
ADAR expression vs. sex. . . . 14
Position of candidate RNA editing events in sequencing reads. . . . 15
Correlation coefficients between paired macrophage and foam cell samples used to evaluate reproducibility. 16

List of supplementary tables

Overview of studied tissues and sequencing . . . 17
No. sequencing reads per tissue before mapping. . . . 18
No. of events called per tissue and library type. . . . 19
mRNA recoding events. . . . 20
Edited microRNAs and snoRNAs. . . . 21
Associations between A-to-G(I) editing and clinical parameters. . . . 22
List of discovered RNA editing events . . . 23
List of identified RNA editing QTLs. . . . 24

1 Supplementary figures

Supplementary Fig. 1. Flowchart of the pipeline used to call RNA editing events. Squares and octagons represent datasets and algorithms/programs, respectively. The beige area indicates the first level of filtering steps, and the blue area indicates the second level of filtering steps (taking place on concordant alignments). The pipeline includes two separate alignment steps employing the programs STAR [1] and GSNAP [2]. Only alignments that are concordant between these two programs are used. Several external datasets are used to remove genomic polymorphisms (dbSNP 141/146/147, Exome Aggregation Consortium variants, NHLBI Exome seq. variants, Scripps Wellderly variants, and COSMIC). In addition, variants within the extended major histocompatibility complex (chr6:28M-33M) are removed. Scripts used to run the pipeline have been deposited on GitHub: https://github.com/oscar-franzen/rnaed

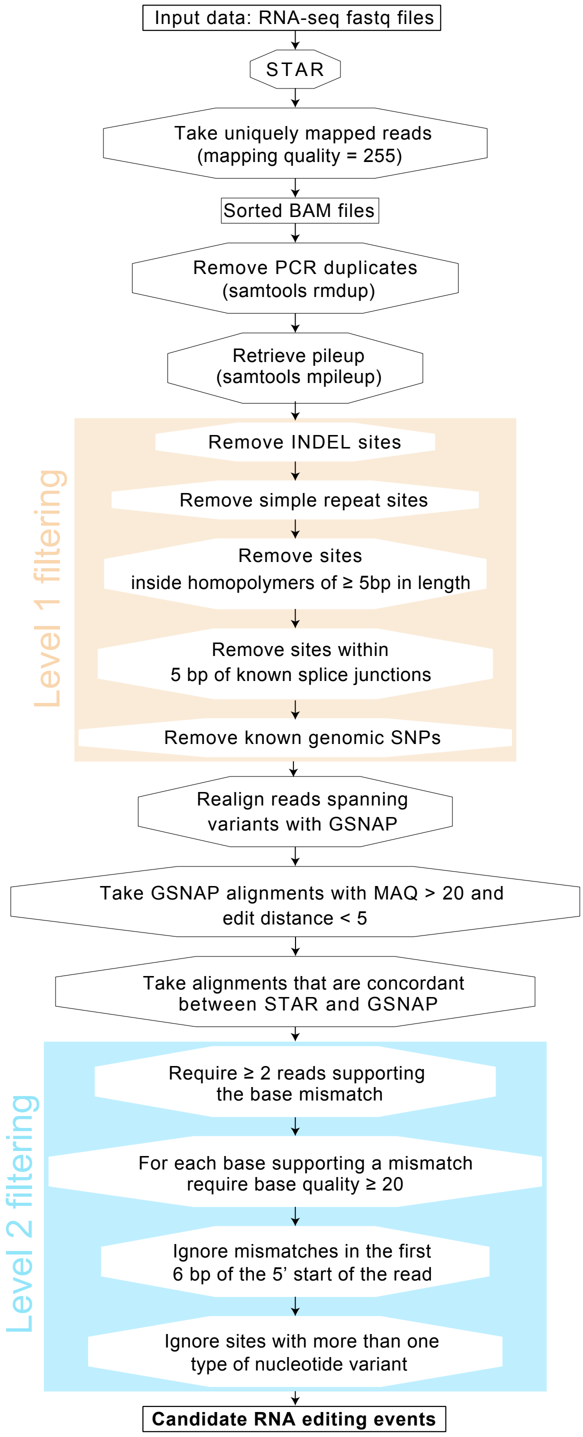

Supplementary Fig. 2. Intersection of A-to-G(I) RNA editing events with public databases. Exact chromosome-position of editing events were compared with events reported in REDIportal [3] and DARNED [4]. Note that the RADAR database [5] is embeded in REDIportal.

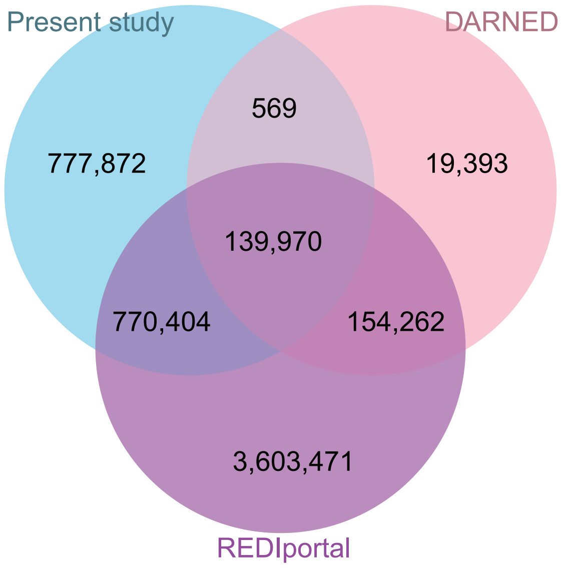

Supplementary Fig. 3. Genomic annotation of single nucleotide variants. The genomic position of the single nucleotide variant was fed into ANNOVAR [6]. The y-axis shows the number of SNV, and the x-axis shows the type of genomic feature. Colors correspond to the type of change; e.g., the canonical RNA editing event A-to-G(I) is red.

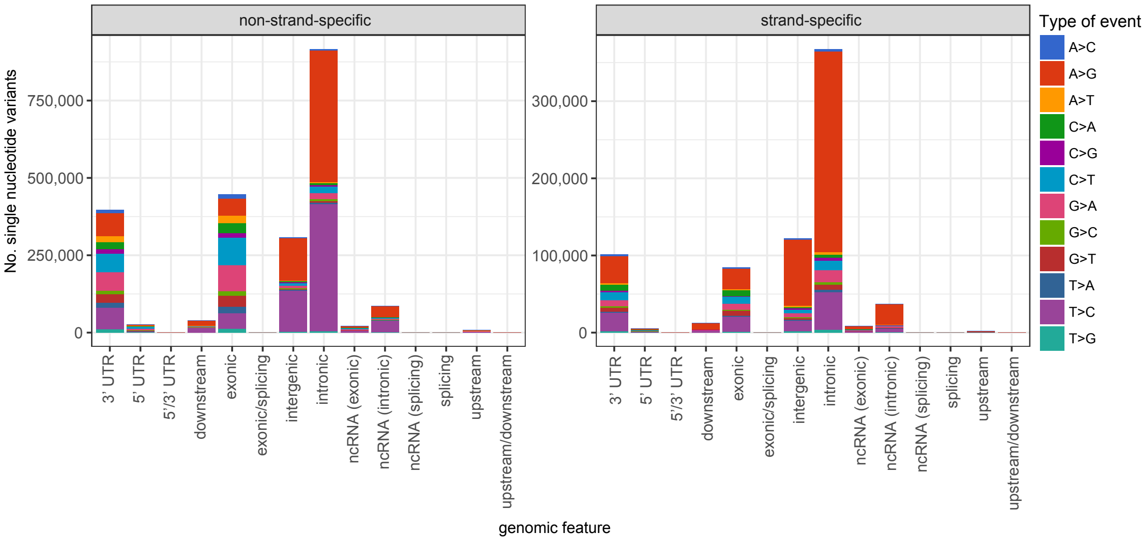

Supplementary Fig. 4. Percent A-to-G(I) events found in different number of tissues. The percent (y-axis) of A-to-G(I) editing events found in 7 tissues (AOR, MAM, BLO, SUF, VAF, LIV, and SKM). Blue color shows the analysis done in all genes regardless of expression. Red color shows the analysis done in genes that are robustly expressed (defined as median(RPKM) > 10 across all samples). The majority, approximately 67-69%, of A-to-G(I) editing events were found in one tissue. Exact chromosome-positions were compared.

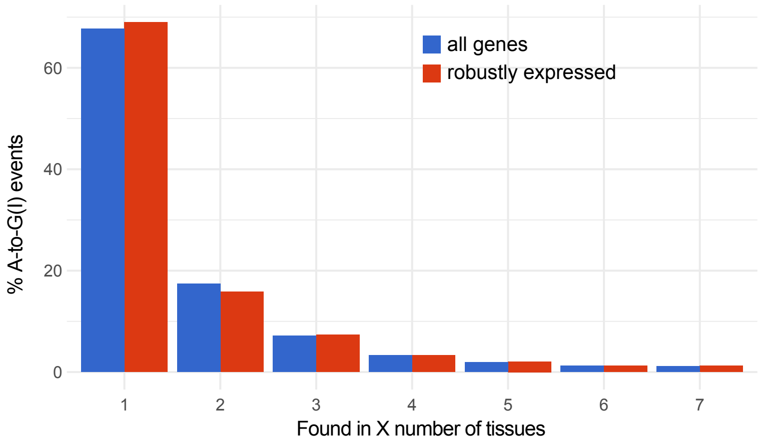

Supplementary Fig. 5. Scatter plots showing the relationship between sequencing depth and number of called RNA editing events. Y- and x-axes show the number of called A-to-G(I) RNA editing events and the sequencing depth (in million uniquely mapped reads after collapsing PCR duplicates), respectively. Each dot represents one tissue sample that has undergone RNA-seq. Colors in each plot correspond to strand-specific and non-strand-specific sequencing, respectively. There is an approximate linear relationship between detection of RNA editing and sequencing depth.

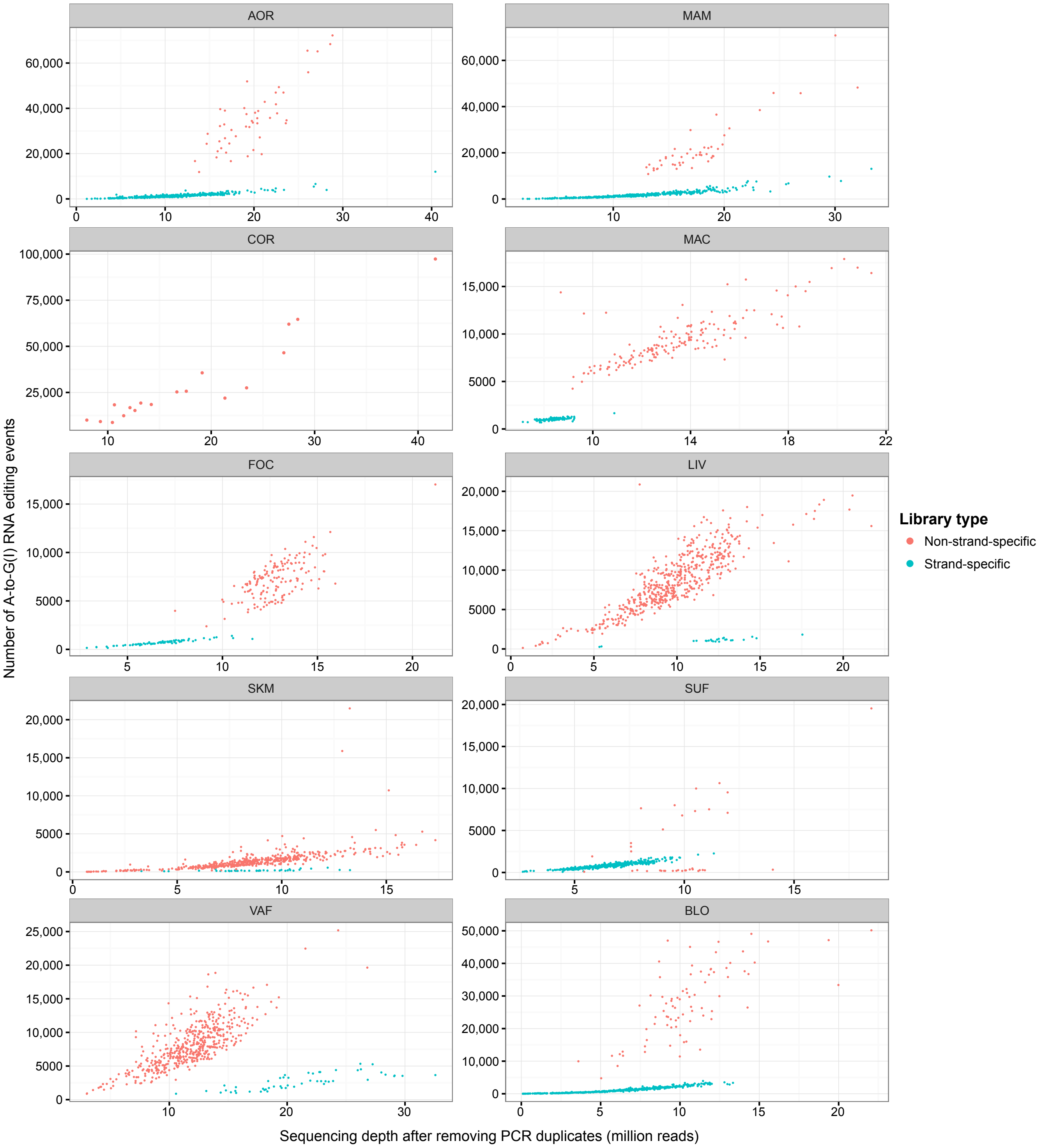

Supplementary Fig. 6. Cumulative number of detected A-to-G(I) RNA editing events and genes. (A) The number of RNA editing events detected with increasing number of samples. Initially, the sample list was randomized and the unique number of events was counted as the number of samples increases. (B) Same concept as (A), but instead showing genes.

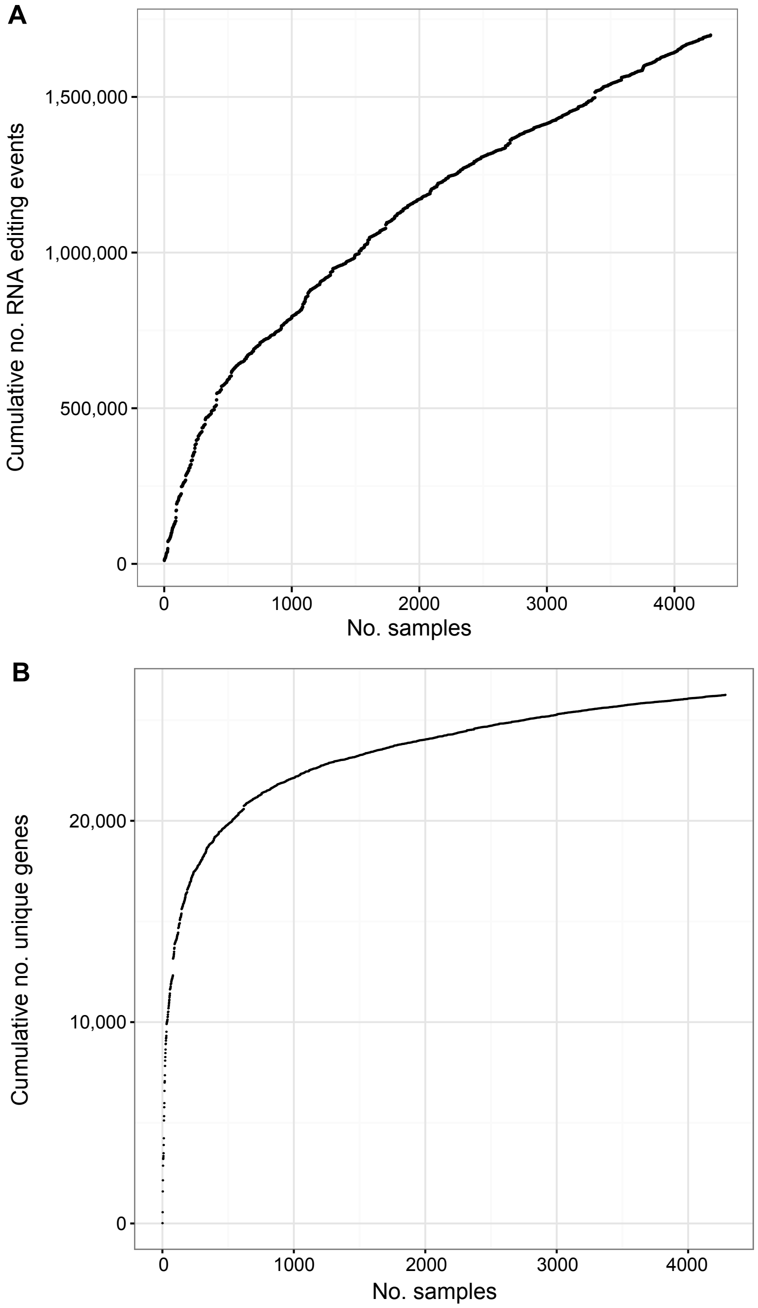

Supplementary Fig. 7. A-to-G(I) events falling in different repeat classes. Percent (y-axis) of A-to-G(I) events in various human repeat classes (x-axis). Red color indicates genome coverage of the repeat.

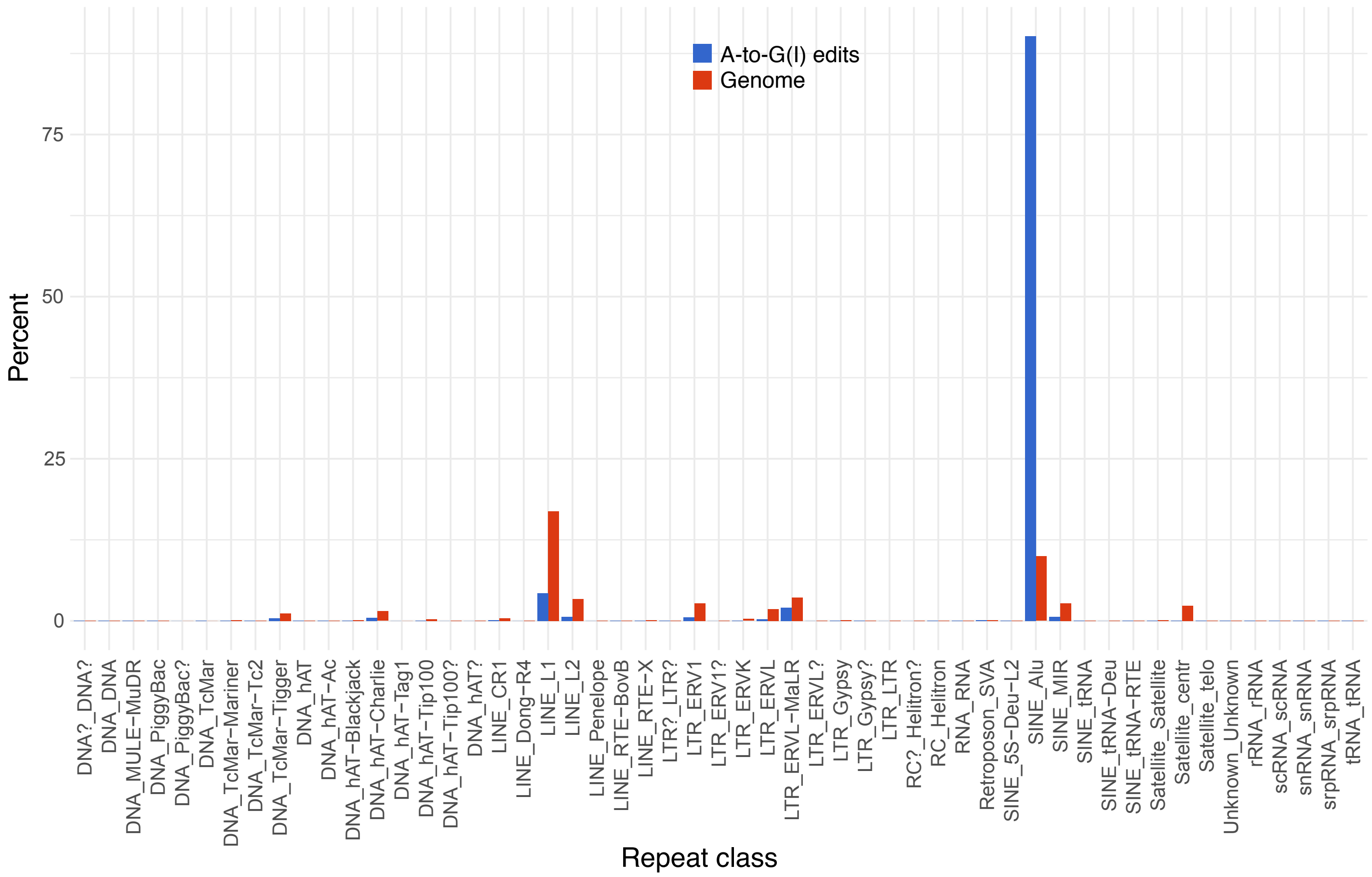

Supplementary Fig. 8. A-to-G(I) events falling in *Alu* subtypes. Percent (y-axis) A-to-G(I) falling in *Alu* subtypes (x-axis) as blue color and the percent of the genome covered by the particular subtype (red color).

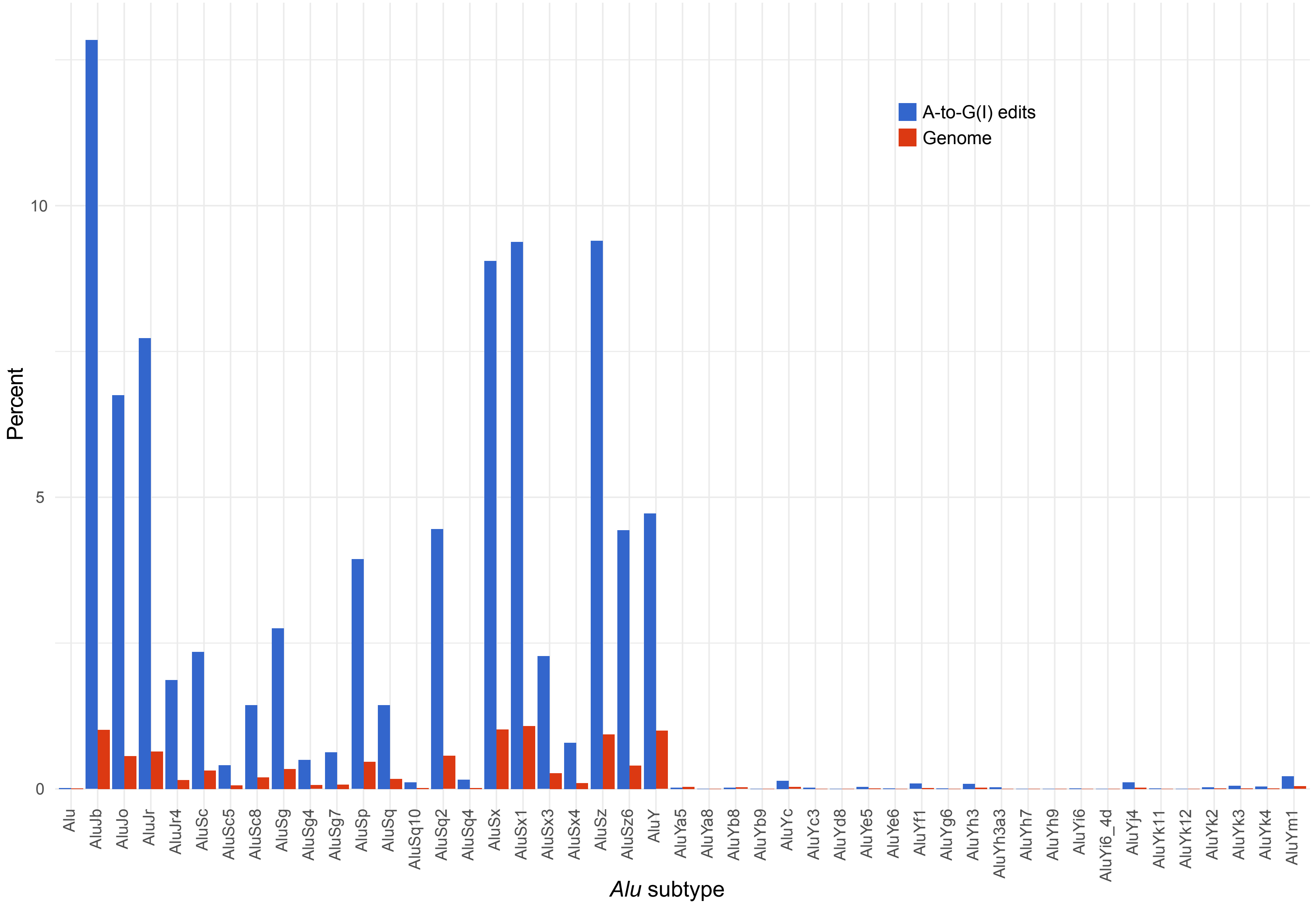

Supplementary Fig. 9. C-to-T(U) events falling in various repeats. Barplot showing distribution of C-to-T(U) editing events inside repeat elements. Blue color refers to percent C-to-T(U) events that fall in any of the examined repeat family/class (x-axis). Red colors refers to percent genome coverage of the repeat family/class.

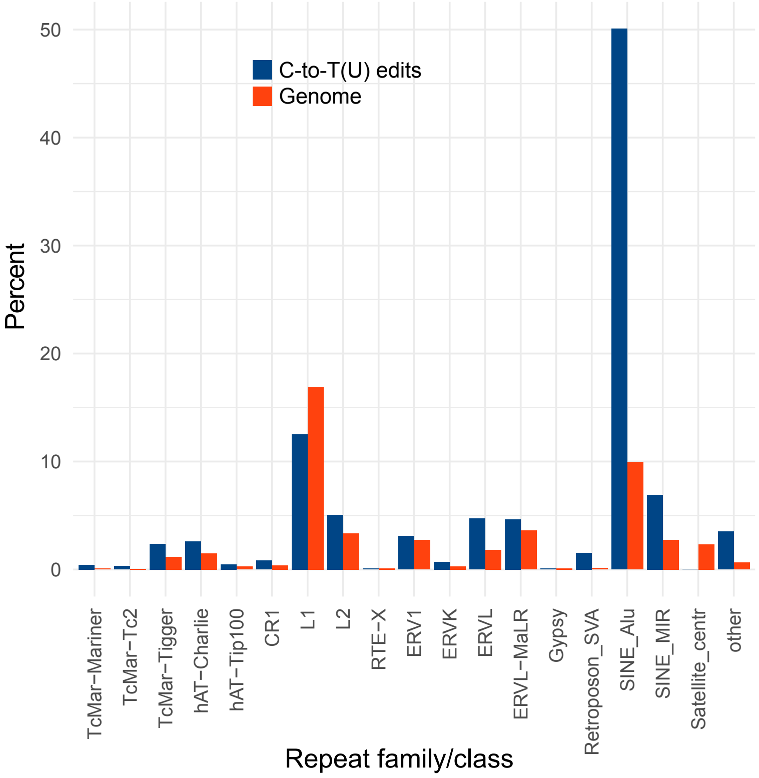

Supplementary Fig. 10. Comparison of *ADAR* expression across tissues. Box-plots of *ADAR* (syn. *ADAR1*), *ADAR2* (syn. *ADARB1*), and *ADAR3* (syn. *ADARB2*) across the studied tissues/cell types. The y-axis shows expression of individual samples in RPKM [7] and the x-axis shows the tissue. The Ensembl [8] identifier is specified within parenthesis. Black dots are outlier samples.

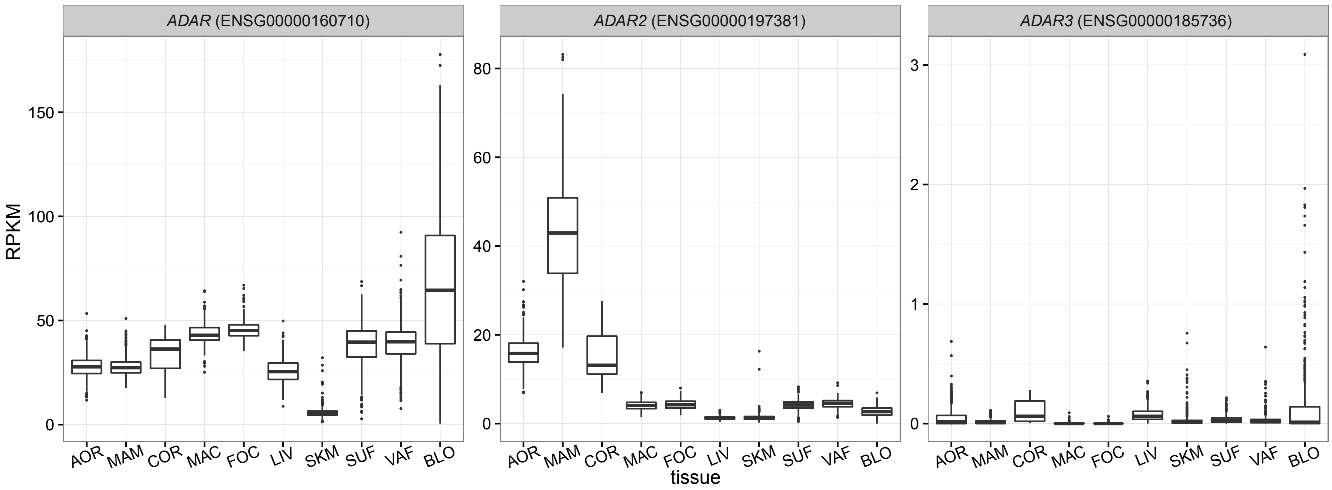

Supplementary Fig. 11. Scatterplot of *ADAR* expression in whole blood versus number of identified A-to-G(I) events. Each dot represents one sample. The number of identified A-to-G(I) events are on the y-axis and *ADAR* (syn. *ADAR1*, ENSG00000160710) expression is shown on the x-axis (RPKM [7]). Only strand-specific samples are shown (n=479). Correlation coefficients are indicated on the top left corner in the plot. There is an approximate linear relationship between *ADAR* expression and number of identified editing events.

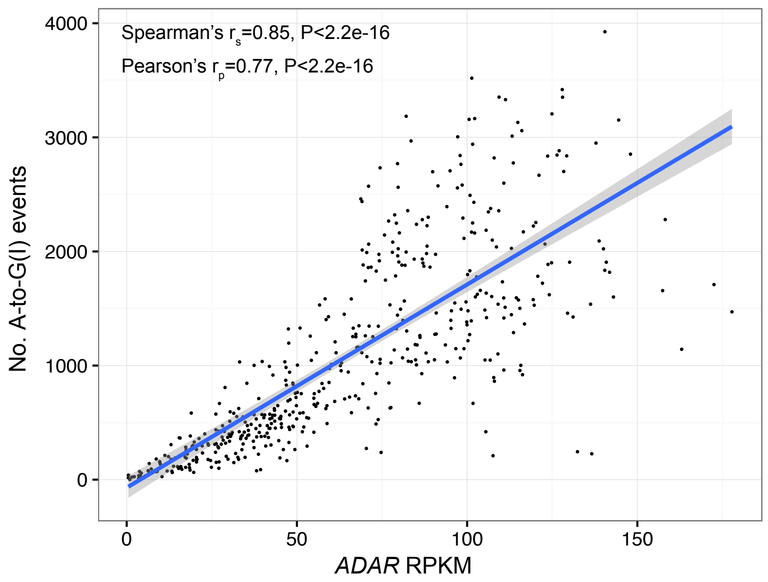

Supplementary Fig. 12. ***ADAR*** **expression vs. sex.** Same as Fig. 10 with the addition of sex stratification. Colors correspond to sex. Significance between female and male was evaluated with Welch's t-test. Abbreviations: N.S. for non-significant, * for P<.05, ** for P<.01, and *** for P<.001

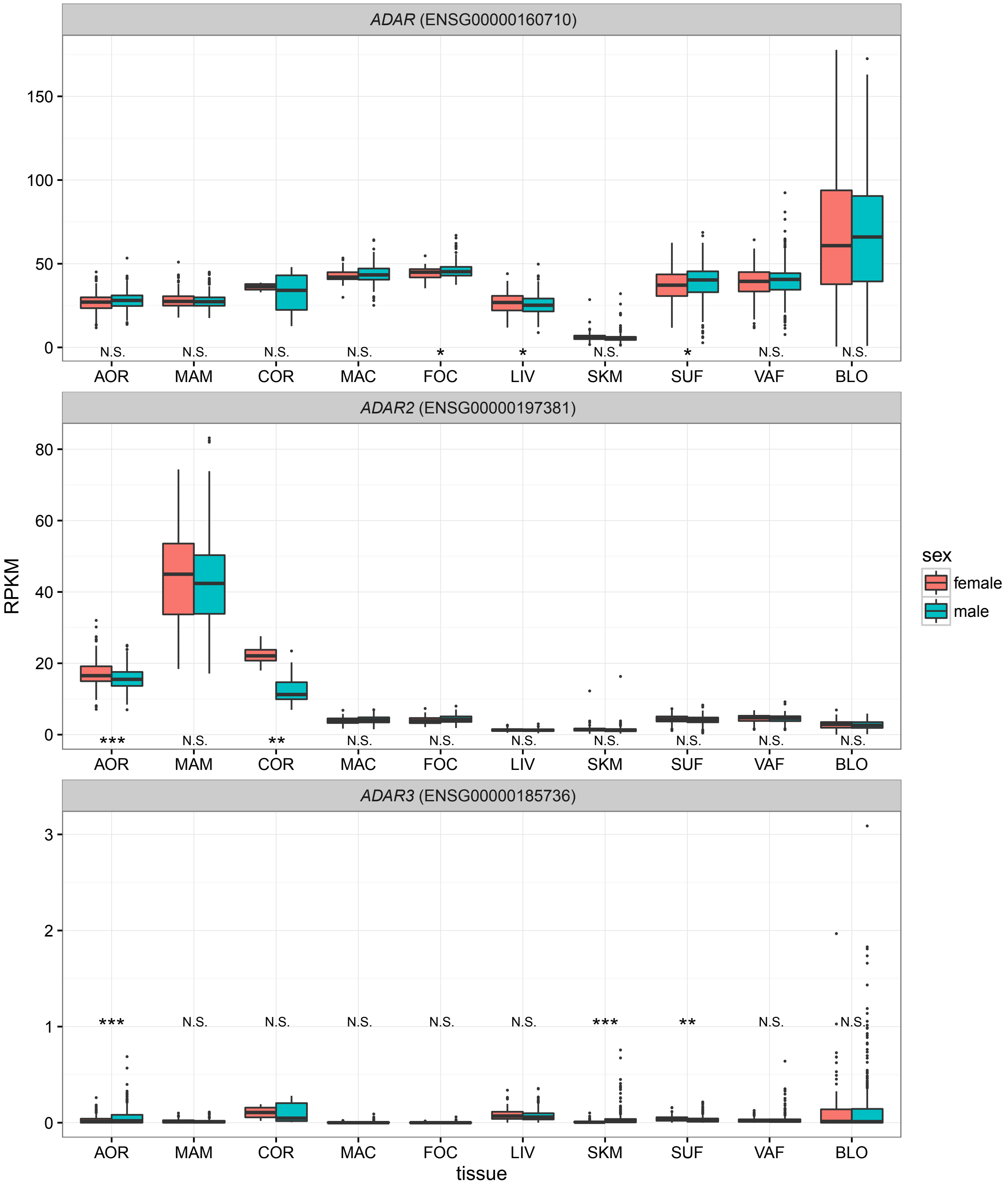

Supplementary Fig. 13. Position of candidate RNA editing events in sequencing reads. Plots are showing where candidate RNA editing events fall on sequencing reads. The x-axis shows the position in reads (from start to end), and the y-axis shows the number of reads harboring a candidate event at this position. Canonical events are indicated in red, and are relatively uniform; i.e., not enriched at start and end positions of sequencing reads, which would suggest major impact of sequencing errors.

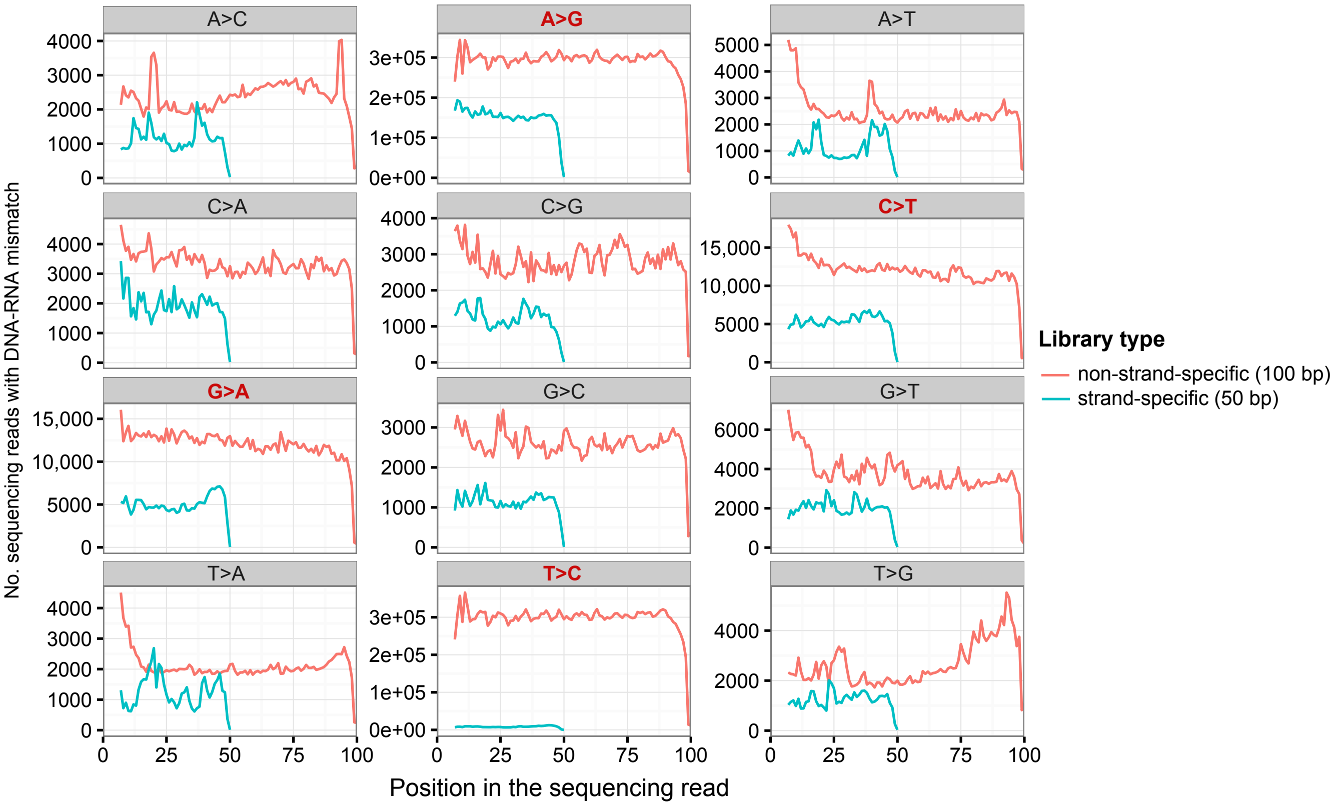

Supplementary Fig. 14. Correlation coefficients between paired macrophage and foam cell samples used to evaluate reproducibility. Boxplot with overlayed jitter showing Spearman's rank correlation coefficients (ρ) for 235 individuals. Each jitter dot is ρ computed from all RNA editing sites detected in common in the pair. Large dots are outlier samples relating to the box-plot.

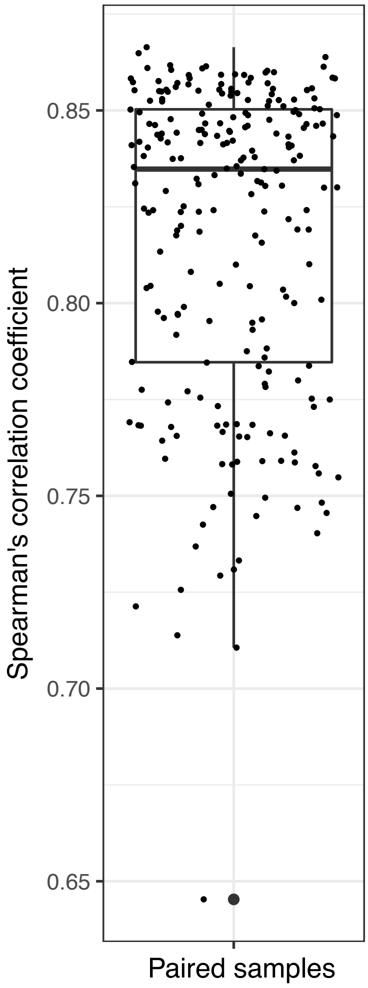

2 Supplementary Tables

Supplementary Table 1. Overview of studied tissues and sequencing. The tissues included in the study. The three-letter abbreviations are used throughout the study. Protocol refers to rRNA-depletion method. Read length is the sequence length in base pairs.

				Number of samples	
**Tissue**	**Abbrev.**	**Protocol**	**Read len. (bp)**	**Strand-spec.**	**Non-strand spec.**
Artery Aorta	`AOR`	poly(A)	100	0	0
		poly(A)	50	0	0
		Ribo-Zero	100	0	47
		Ribo-Zero	50	491	0
Internal Mammary artery	`MAM`	poly(A)	100	0	0
		poly(A)	50	0	0
		Ribo-Zero	100	0	47
		Ribo-Zero	50	505	0
Coronary artery	`COR`	poly(A)	100	0	18
		poly(A)	50	0	0
		Ribo-Zero	100	0	0
		Ribo-Zero	50	0	0
Macrophages	`MAC`	poly(A)	100	0	158
		poly(A)	50	98	0
		Ribo-Zero	100	0	0
		Ribo-Zero	50	0	0
Foam cells	`FOC`	poly(A)	100	0	152
		poly(A)	50	83	0
		Ribo-Zero	100	0	0
		Ribo-Zero	50	0	0
Liver	`LIV`	poly(A)	100	0	521
		poly(A)	50	0	0
		Ribo-Zero	100	0	0
		Ribo-Zero	50	24	0
Skeletal muscle	`SKM`	poly(A)	100	6	493
		poly(A)	50	0	0
		Ribo-Zero	100	0	0
		Ribo-Zero	50	34	0
Subcutaneous fat	`SUF`	poly(A)	100	0	37
		poly(A)	50	495	0
		Ribo-Zero	100	0	0
		Ribo-Zero	50	0	0
Visceral fat	`VAF`	poly(A)	100	16	481
		poly(A)	50	1	0
		Ribo-Zero	100	0	1
		Ribo-Zero	50	34	0
Whole blood	`BLO`	poly(A)	100	3	0
		poly(A)	50	477	0
		Ribo-Zero	100	0	79
		Ribo-Zero	50	0	0
Total				2267	2034

Supplementary Table 2. No. sequencing reads per tissue before mapping. Total refers to the sum over all samples. Median refers to the sample median.

**Tissue**	**Library type**	Total	Median
AOR	Non strand-specific	1,842,126,060	37,664,582
AOR	Strand-specific	10,609,649,917	20,705,888
MAM	Non strand-specific	1,843,931,345	36,937,519
MAM	Strand-specific	14,175,102,166	28,333,577
COR	Non strand-specific	1,065,691,792	47,522,172
COR	Strand-specific	0	0
MAC	Non strand-specific	6,211,276,983	37,995,422
MAC	Strand-specific	3,451,614,978	34,791,928
FOC	Non strand-specific	5,407,123,613	34,831,224
FOC	Strand-specific	2,117,875,052	25,459,217
LIV	Non strand-specific	18,186,253,276	34,970,064
LIV	Strand-specific	663,211,152	27,891,564
SKM	Non strand-specific	18,617,031,950	37,191,457
SKM	Strand-specific	1,530,506,190	32,297,394
SUF	Non strand-specific	1,277,331,297	31,295,378
SUF	Strand-specific	14,921,737,435	30,009,616
VAF	Non strand-specific	17,612,320,995	36,228,285
VAF	Strand-specific	2,578,900,776	52,327,185
BLO	Non strand-specific	2,799,055,660	32,552,368
BLO	Strand-specific	14,673,603,241	30,622,274

Supplementary Table 3. No. of events called per tissue and library type. The table gives the total number of candidate RNA editing events for the twelve possible editing types across all samples for every tissue-library combination. Note, for each cell, if the same site is found multiple times then it is only counted once.

Tissue	Library type	A>C	A>G	A>T	C>A	C>G	C>T	G>A	G>C	G>T	T>A	T>C	T>G
AOR	non-strand-specific	1236	247,550	1144	1526	1362	5204	5187	1403	1584	1147	235,321	1329
AOR	strand-specific	1789	176,458	2361	2323	2375	8628	12,048	1903	4912	2324	34,501	3143
BLO	non-strand-specific	1100	210,427	878	1426	1182	5536	5221	1224	1661	876	213,976	1025
BLO	strand-specific	1642	76,680	1890	3419	1644	8296	6910	1967	2684	1443	5633	1302
COR	non-strand-specific	906	111,274	673	652	884	2551	2352	843	735	694	107,496	914
FOC	non-strand-specific	5124	71,129	4529	3489	3348	12,624	11,580	3389	4057	4345	65,795	5065
FOC	strand-specific	963	12,098	220	8725	305	1822	1261	347	380	217	1023	293
LIV	non-strand-specific	10,939	175,646	11,094	12,089	8398	56,988	53,141	8216	14,986	8710	167,572	11,052
LIV	strand-specific	131	15,879	160	160	179	582	818	131	268	169	2209	177
MAC	non-strand-specific	2229	101,186	5997	9491	5657	19,170	18,583	5499	9954	6015	98,705	2279
MAC	strand-specific	324	17,869	355	551	465	3491	1835	516	553	295	1396	383
MAM	non-strand-specific	1288	168,319	1107	1351	1422	4943	4766	1434	1452	1008	163,427	1393
MAM	strand-specific	2282	229,323	2817	3221	2699	11,151	14,161	2519	7971	2956	43,880	3188
SKM	non-strand-specific	4793	71,981	16,592	23,843	7246	54,470	53,124	7152	25,033	16,115	68,859	4947
SKM	strand-specific	175	6412	278	250	200	1099	1301	215	612	230	3333	259
SUF	non-strand-specific	751	24,875	739	921	658	6044	5469	670	1333	541	24,948	765
SUF	strand-specific	2751	46,327	1547	4780	1655	10,585	6447	2277	4939	1186	5437	1347
VAF	non-strand-specific	12,265	184,731	16,122	20,716	16,625	59,600	56,901	16,319	23,594	13,981	179,496	12,510
VAF	strand-specific	697	74,127	877	1097	938	3183	3827	763	2280	882	29,109	964

Supplementary Table 4. mRNA recoding events. Rows colored blue indicate novel events (not reported in REDIportal, DARNED, nor found to be published elsewhere).

								Number of samples[4]									Median editing ratio[4]								
chr.	pos. (GRCh38)	gene	description	REDIportal[1]	DARNED[1]	change[2]	ref.[3]	AOR	BLO	FOC	LIV	MAM	MAC	SUF	SKM	VAF	AOR	BLO	FOC	LIV	MAM	MAC	SUF	SKM	VAF
1	109,713,682	*GSTM5*	Glutathione S-transferase mu 5	Y	Y	NM_000851:K94R	-	-	-	-	-	-	-	24	-	-	-	-	-	-	-	-	0.16	-	-
1	155,309,905	*FDPS*	Farnesyl Diphosphate Synthase	Y	N	NM_001135821:Y39C	-	-	-	-	26	-	-	-	-	-	-	-	-	0.11	-	-	-	-	-
1	225,786,912	*SRP9*	Signal Recognition Particle 9	Y	Y	NM_001130440:I64M	[9]	84	77	152	496	143	159	54	308	475	0.78	0.46	0.29	0.36	0.76	0.28	0.45	0.20	0.53
1	225,786,943	*SRP9*	Signal Recognition Particle 9	Y	N	NM_001130440:S75G	-	-	59	125	369	-	145	107	-	364	-	0.25	0.12	0.14	-	0.11	0.18	-	0.11
2	108,298,644	*SULT1C2*	Sulfotransferase 1C2	Y	N	NM_176825:S119G	-	-	-	21	-	-	-	-	-	-	-	-	0.17	-	-	-	-	-	-
2	219,483,602	*SPEG*	SPEG complex locus	N	N	NM_005876:S2047G	[10][5]	48	-	-	-	44	-	-	-	-	0.57	-	-	-	0.81	-	-	-	-
3	49,360,949	*RHOA*	Ras homolog gene family, member A	Y	Y	NM_001313943:R176G	-	-	45	27	46	-	27	-	-	96	-	0.50	0.40	0.50	-	0.50	-	-	0.32
3	49,360,951	*RHOA*	Ras homolog gene family, member A	Y	Y	NM_001313943:Y175C	-	-	-	-	29	-	-	-	-	47	-	-	-	0.50	-	-	-	-	0.33
3	49,360,961	*RHOA*	Ras homolog gene family, member A	Y	Y	NM_001313943:S172G	-	-	-	-	-	-	-	-	-	27	-	-	-	-	-	-	-	-	0.33
3	49,360,990	*RHOA*	Ras homolog gene family, member A	Y	Y	NM_001313943:K162R	-	-	45	24	49	-	28	-	-	57	-	0.50	0.33	0.50	-	0.33	-	-	0.25
3	179,375,226	*MFN1*	Mitofusin-1	Y	N	NM_033540:I328V	[10][5]	-	-	-	-	39	-	-	-	-	-	-	-	-	0.15	-	-	-	-
4	2,938,299	*NOP14*	NOP14 Nucleolar Protein	Y	Y	NM_001291979:I779V	-	-	97	70	380	23	89	95	-	299	-	0.30	0.23	0.27	0.50	0.18	0.27	-	0.19
4	2,938,304	*NOP14*	NOP14 Nucleolar Protein	Y	Y	NM_001291979:Q777R	-	-	-	-	27	-	-	-	-	25	-	-	-	0.17	-	-	-	-	0.12
4	57,110,062	*IGFBP7*	Insulin Like Growth Factor Bind. Prot. 7	N	N	NM_001553:K97R	[11][6]	-	-	-	-	208	-	-	-	-	-	-	-	-	0.11	-	-	-	-
4	57,110,146	*IGFBP7*	Insulin Like Growth Factor Bind. Prot. 7	N	N	NM_001553:E69G	-	50	-	-	-	76	-	-	-	-	0.12	-	-	-	0.16	-	-	-	-
4	157,336,723	*GRIA2*	Glut. Ionotropic Rec. AMPA Subunit 2	Y	Y	NM_001083620:Q560R,NM_001083619:Q607R	[12]	252	-	-	-	136	-	-	-	-	1.00	-	-	-	1.00	-	-	-	-
5	38,949,393	*RICTOR*	Rapamycin-insensitive companion of mTOR	Y	N	NM_001285439:R1391G	-	137	-	-	-	258	-	-	-	-	0.56	-	-	-	0.67	-	-	-	-
5	178,135,225	*RMND5B*	Req. For Meiotic Nuclear Div. 5 Hom. B	Y	N	NM_001288795:S5G	[13]	-	-	-	-	-	-	-	-	38	-	-	-	-	-	-	-	-	0.67
5	178,135,267	*RMND5B*	Req. For Meiotic Nuclear Div. 5 Hom. B	Y	N	NM_001288795:S19G	[13]	-	-	-	-	-	-	-	-	41	-	-	-	-	-	-	-	-	0.60
6	33,788,465	*LEMD2*	LEM Domain Containing 2	Y	N	NM_181336:S218G	-	-	-	-	-	33	-	-	-	-	-	-	-	-	0.18	-	-	-	-
6	44,152,612	*TMEM63B*	Transmembrane Protein 63B	Y	N	NM_018426:Q619R	[14]	-	-	-	-	23	-	-	-	-	-	-	-	-	0.67	-	-	-	-
7	38,262,191	*TARP*	TCR gamma alt. reading frame prot.	N	N	NM_001003806:N58S	-	-	29	-	-	-	-	-	-	-	-	0.27	-	-	-	-	-	-	-
7	39,950,928	*CDK13*	Cyclin-Dependent Kinase 13	Y	N	NM_003718:K96R	[11]	22	63	-	-	-	-	-	-	-	0.50	0.24	-	-	-	-	-	-	-
7	131,510,304	*PODXL*	Podocalyxin Like	Y	N	NM_001018111:Q245R	-	-	-	-	-	-	-	-	-	26	-	-	-	-	-	-	-	-	0.11
7	131,510,308	*PODXL*	Podocalyxin Like	Y	N	NM_001018111:S244G	-	-	-	-	-	-	-	-	-	67	-	-	-	-	-	-	-	-	0.13
7	131,510,316	*PODXL*	Podocalyxin Like	Y	N	NM_001018111:H241R	[15]	-	-	-	-	-	-	-	26	188	-	-	-	-	-	-	-	0.40	0.17
8	144,247,668	*MROH1*	Maestro Heat Like Rep. Fam. Mem. 1	N	N	NM_032450:S1037G,NM_001288814:S1028G	-	-	-	-	26	-	-	-	-	28	-	-	-	0.50	-	-	-	-	0.45
9	33,271,197	*CHMP5*	chromatin-mod. prot./charged multives. body prot.	N	N	NM_001195536:K121E	-	-	117	-	-	-	-	188	-	-	-	0.17	-	-	-	-	0.12	-	-
9	130,114,583	*GPR107*	G protein-coupled receptor 107	Y	N	NM_001136557:H457R	[16]	-	42	-	-	-	-	-	-	-	-	0.60	-	-	-	-	-	-	-
9	130,114,595	*GPR107*	G protein-coupled receptor 107	Y	N	NM_001136557:Q461R	[16]	-	33	-	-	-	-	-	-	-	-	0.50	-	-	-	-	-	-	-
10	45,789,442	*FAM21C*	Fam. w/ Seq. Sim. 21 Member C	N	N	NM_001169106:K1158R,NM_001169107:K1124R,NM_015262:K1199R	-	-	30	-	31	-	-	33	29	34	-	0.55	-	0.47	-	-	0.46	0.46	0.54
10	95,387,021	*SORBS1*	Sorbin And SH3 Domain Containing 1	Y	N	NM_001034955:T466A	-	28	-	-	-	-	-	-	-	-	0.23	-	-	-	-	-	-	-	-
10	95,387,064	*SORBS1*	Sorbin And SH3 Domain Containing 1	Y	N	NM_001034955:I451M	-	39	-	-	-	35	-	-	47	-	0.33	-	-	-	0.33	-	-	0.50	-
10	95,387,072	*SORBS1*	Sorbin And SH3 Domain Containing 1	Y	N	NM_001034955:T449A	-	41	-	-	-	34	-	-	64	-	0.40	-	-	-	0.33	-	-	0.57	-
10	100,924,268	*SLF2*	SMC5-SMC6 Complex Loc. Factor 2	Y	N	NM_001136123:S423G	[17]	-	-	-	-	27	-	-	-	-	-	-	-	-	0.12	-	-	-	-
10	124,762,463	*METTL10*	Methyltransferase Like 10	Y	Y	NM_001304467:T160A,NM_212554:T238A	[11]	-	-	-	-	-	-	-	-	57	-	-	-	-	-	-	-	-	0.17
10	124,762,529	*METTL10*	Methyltransferase Like 10	Y	Y	NM_001304467:T138A,NM_212554:T216A	-	-	-	-	-	-	-	-	-	34	-	-	-	-	-	-	-	-	0.19
10	133,297,335	*TUBGCP2*	Tubulin Gamma Complex Assoc. Prot. 2	Y	Y	NM_001256617:N229S	-	26	46	58	277	-	62	-	29	324	0.82	0.50	0.50	0.67	-	0.43	-	0.50	0.86
10	133,297,336	*TUBGCP2*	Tubulin Gamma Complex Assoc. Prot. 2	Y	N	NM_001256617:N229D	-	-	-	-	47	-	-	-	-	-	-	-	-	0.33	-	-	-	-	-
10	133,297,396	*TUBGCP2*	Tubulin Gamma Complex Assoc. Prot. 2	Y	N	NM_001256617:R209D	-	-	-	-	93	-	-	-	-	29	-	-	-	0.33	-	-	-	-	0.33
12	11,034,879	*PRH1-TAS2R14*	readthrough transcript encoding a fusion protein	Y	Y	NM_001316893:S15G	-	-	-	-	-	21	-	-	-	-	-	-	-	-	0.22	-	-	-	-
12	57,625,434	*SLC26A10*	Solute Carrier Fam. 26 Mem. 10	N	N	NM_133489:R500G	-	-	-	-	-	40	-	-	-	-	-	-	-	-	0.54	-	-	-	-
12	132,862,348	*CHFR*	E3 Ubiquitin Protein Ligase	Y	N	NM_018223:S161G	-	-	35	34	-	-	50	-	-	41	-	0.67	0.33	-	-	0.33	-	-	0.50
12	132,862,363	*CHFR*	E3 Ubiquitin Protein Ligase	Y	N	NM_018223:T156A	-	34	-	-	-	23	36	-	-	111	0.71	-	-	-	1.00	0.29	-	-	0.67
13	45,516,236	*COG3*	Comp. Of Oligomeric Golgi Complex 3	Y	Y	NM_031431:I635V	[9]	122	130	164	326	143	178	217	49	386	0.74	0.19	0.29	0.25	0.67	0.29	0.67	0.25	0.58
15	64,957,127	*ANKDD1A*	Ankyrin Rep. and Death Domain Cont. 1A	Y	N	NM_182703:Q503R	[11]	-	-	-	-	-	-	-	-	31	-	-	-	-	-	-	-	-	0.20
16	5,044,722	*C16orf89*		Y	N	NM_152459:Y357C	-	-	-	-	-	-	-	-	-	44	-	-	-	-	-	-	-	-	0.50
16	5,044,732	*C16orf89*		Y	N	NM_152459:S354G	-	-	-	-	-	-	-	-	-	23	-	-	-	-	-	-	-	-	0.50
16	30,188,879	*CORO1A*	Coronin 1A	N	N	NM_001193333:E434G	-	-	-	-	-	-	-	-	-	22	-	-	-	-	-	-	-	-	0.17
16	57,683,958	*ADGRG3*	Adhesion G Prot.-Coupled Rec. G3	Y	N	NM_170776:E303G,NM_001308360:E183G	-	-	51	-	-	-	-	-	-	-	-	0.11	-	-	-	-	-	-	-
17	1,534,142	*PITPNA*	Phosphatidylinositol Transfer Prot. Alpha	N	N	NM_006224:D242G	-	-	-	-	24	-	-	-	-	-	-	-	-	0.10	-	-	-	-	-
19	7,520,407	*ZNF358*	Zinc Finger Protein 358	N	N	NM_018083:S389G	-	-	-	-	-	39	-	-	-	-	-	-	-	-	0.25	-	-	-	-
19	14,482,793	*GIPC1*	GIPC PDZ domain cont. fam., mem. 1	Y	N	NM_202468:T62A	[18]	-	-	-	-	50	-	-	-	-	-	-	-	-	0.29	-	-	-	-
19	42,515,077	*CEACAM1*	Carcinoembryonic Antigen Rel. Cell Adhesion Mol. 1	Y	N	NM_001184815:R329G	-	-	-	-	33	-	-	-	-	-	-	-	-	0.67	-	-	-	-	-
19	46,338,233	*HIF3A*	Hypoxia Inducible Factor 3 Alpha Subunit	Y	N	NM_152796:H427R	-	-	-	-	-	-	-	-	-	35	-	-	-	-	-	-	-	-	0.40
19	46,338,281	*HIF3A*	Hypoxia Inducible Factor 3 Alpha Subunit	Y	N	NM_152796:Q443R	-	-	-	-	-	-	-	27	-	43	-	-	-	-	-	-	0.50	-	0.43
19	46,649,597	*DACT3*	dishevelled bind. antagonist of beta catenin 3	Y	N	NM_145056:R259G,NM_001301046:R34G	[19]	28	-	-	-	32	-	-	-	-	0.50	-	-	-	0.58	-	-	-	-
19	54,221,229	*LILRB3*	Leukocyte Immunoglobulin Like Receptor B3	N	N	NM_001081450:Q270R	-	-	-	106	-	-	132	-	-	-	-	-	0.23	-	-	0.21	-	-	-
19	54,221,256	*LILRB3*	Leukocyte Immunoglobulin Like Receptor B3	N	N	NM_001081450:E261G	-	-	-	88	-	-	126	-	-	-	-	-	0.53	-	-	0.50	-	-	-
19	57,686,495	*ZNF551*	Zinc Finger Protein 551	Y	Y	NM_001270938:M46V,NM_138347:M74V	[19]	-	-	-	-	27	-	-	-	-	-	-	-	-	0.67	-	-	-	-
19	57,844,302	*ZNF587B*	Zinc Finger Protein 587B	Y	Y	NM_001204818:K390R	[20]	-	-	-	21	-	-	-	-	21	-	-	-	0.40	-	-	-	-	0.29
19	57,844,321	*ZNF587B*	Zinc Finger Protein 587B	Y	Y	NM_001204818:I396M	[20]	32	71	48	179	28	40	24	22	217	0.75	0.67	0.67	0.67	0.67	0.67	1.00	0.50	0.62
20	5,111,526	*TMEM230*	Transmembrane Protein 230	Y	Y	NM_001009923:S50G	-	-	-	-	22	-	-	-	-	21	-	-	-	0.21	-	-	-	-	0.12
20	5,111,540	*TMEM230*	Transmembrane Protein 230	Y	Y	NM_001009923:H45R	[21]	-	-	-	27	-	-	-	-	219	-	-	-	0.29	-	-	-	-	0.25
20	37,519,161	*BLCAP*	Bladder Cancer Associated Protein	Y	Y	NM_001167823:Q5R	[22]	28	-	-	-	35	-	39	-	-	0.13	-	-	-	0.12	-	0.13	-	-

[1] If the change has previously been reported in REDIportal [3] or DARNED [4]. Abbreviations: (Y)es, (N)o.
[2] The primary mRNA and the corresponding amino acid change as: [amino acid encoded in the genome][primary sequence position][amino acid resulting from RNA editing event]. Can be more than one if more than one isoform is affected.
[3] Reference if the recoding event was previously reported in the literature.
[4] A dash in the cell indicates that the RNA editing event did not satisfy the detection criteria for the particular tissue.
[5] Danecek *et al.* found the change in mouse.
[6] Supplementary Data 4.

Supplementary Table 5. Edited microRNAs and snoRNAs.

chr.	pos. (GRCh38)	mi/snoRNA ID[1]	REDIportal[2]	DARNED[2]	ref.	Number of samples									Median editing ratio								
						AOR	BLO	FOC	LIV	MAM	MAC	SUF	SKM	VAF	AOR	BLO	FOC	LIV	MAM	MAC	SUF	SKM	VAF
2	69,103,688	pri-mir-3126	N	N		15	0	0	0	44	0	0	0	0	0.4	0	0	0	0.4	0	0	0	0
2	233,288,786	U88 (snoRNA)	N	N		13	1	0	0	50	0	0	0	0	0.047	0.02	0	0	0.06	0	0	0	0
2	233,288,843	U88 (snoRNA)	Y	N		14	22	0	1	18	0	0	0	1	0.039	0.025	0	0.2	0.04	0	0	0	0.15
4	10,078,630	mir-3138	Y	N		32	0	0	0	40	0	0	0	0	1	0	0	0	1	0	0	0	0
7	92,204,095	pri-mir-1285-1	Y	Y		3	2	1	37	1	0	1	1	25	0.66	0.75	1	1	1	0	1	0.66	0.66
9	95,085,457	pri-mir-27b	Y	N		92	0	0	1	191	0	1	1	0	0.30	0	0	0.28	0.5	0	0.5	0.25	0
10	51,299,578	pri-mir-605	Y	N		79	0	0	0	112	0	0	0	0	0.72	0	0	0	1	0	0	0	0
10	51,299,590	mir-605-5p	N	N		26	0	0	0	28	0	0	0	1	0.17	0	0	0	0.36	0	0	0	1
10	51,299,626	mir-605-3p	Y	N		67	0	0	0	53	0	0	0	0	0.61	0	0	0	0.6	0	0	0	0
10	51,299,636	mir-605-3p	N	N		60	0	0	0	68	0	0	0	0	0.2	0	0	0	0.53	0	0	0	0
10	51,299,642	mir-605-3p	Y	Y		145	0	0	0	180	0	0	0	0	0.66	0	0	0	0.75	0	0	0	0
10	51,299,653	pri-mir-605	N	N		39	0	0	0	55	0	0	0	0	0.17	0	0	0	0.4	0	0	0	0
10	68,759,390	pri-mir-1254	Y	Y		20	18	17	83	21	25	2	11	75	0.61	0.66	0.5	0.5	0.66	0.4	1	0.66	0.5
11	93,733,708	pri-mir-1304	Y	Y		22	71	20	155	17	25	5	5	84	0.31	0.66	0.45	0.5	0.33	0.33	1	0.28	0.33
19	13,836,290	mir-24-2-3p	N	N		49	0	0	0	38	0	0	1	1	0.22	0	0	0	0.27	0	0	0.3	0.4
19	13,836,514	pri-mir-27a	Y	N		30	52	0	3	30	0	3	5	0	0.16	0.27	0	0.66	0.30	0	0.25	0.4	0
19	40,282,634	pri-mir-641	Y	N		4	2	0	36	3	1	2	0	24	0.45	1	0	0.5	0.66	0.5	1	0	0.5
20	17,962,796	SNORD17 (snoRNA)	Y	N		57	0	0	0	168	0	1	1	0	0.15	0	0	0	0.2	0	0.5	0.15	0

[1] 'pri' denotes if the editing event is falling within the predicted pri-miRNA sequence.
[2] If the change has previously been reported in REDIportal [3] or DARNED [4]. Abbreviations: (Y)es, (N)o.

Supplementary Table 6. Associations between A-to-G(I) editing and clinical parameters.
This table is given as an external Excel file.

Supplementary Dataset 1. List of discovered RNA editing events. The table is tab separated. The columns correspond to: (1) three letter tissue abbreviation; (2) library type (N=non-strand-specific, Y=strand-specific); (3) chromosome; (4) position; (5) DNA base; (6) RNA base; and (7) number of samples in this tissue-library combination where this event was detected. Coordinates are in GRCh38. This table is given as an external file.

Supplementary Dataset 2. List of identified RNA editing QTLs. The table is tab separated. The columns correspond to: (1) three letter tissue abbreviation; (2) regulatory SNP; (3) the encoded (effect) allele of the regulatory SNP; (4) genomic coordinate of the RNA editing site (GRCh38); (5) molecular interaction type (*cis*=the regulatory SNP and the editing site are on same chromosome, *trans*=the regulatory SNP and the editing site are on different chromosomes); (6) beta coefficient; (7) p-value of the association between RNA editing and the regulatory SNP; (8) adjusted (permutation-based) p-value; (9) the gene overlapping the RNA editing site; (10) same as previous, but giving the gene symbol instead; (11) gene biotype according to GENCODE; (12) the repeat type, if any, overlapping the RNA editing site; (13) the trait, if this regulatory SNP is a reported GWAS lead SNP; (14) gene region; (15) if the gene has previously reported as involved in cardiometabolic traits; (16) if the RNA editing site has been reported in DARNED; (17) if the RNA editing site has been reported in REDIportal; and (18) if the overlapping gene has an eQTL. Abbreviations: (Y)es, (N)o. This table is given as an external file.

3 References

[1] A. Dobin, C. A. Davis, F. Schlesinger, J. Drenkow, C. Zaleski, S. Jha, P. Batut, M. Chaisson, and T. R. Gingeras. STAR: ultrafast universal RNA-seq aligner. *Bioinformatics (Oxford, England)* 29.1 (2013), pp. 15–21. DOI: 10.1093/bioinformatics/bts635.

[2] T. D. Wu and S. Nacu. Fast and SNP-tolerant detection of complex variants and splicing in short reads. *Bioinformatics (Oxford, England)* 26.7 (2010), pp. 873–81. DOI: 10.1093/bioinformatics/btq057.

[3] E. Picardi, A. M. D'Erchia, C. Lo Giudice, and G. Pesole. REDIportal: a comprehensive database of A-to-I RNA editing events in humans. *Nucleic acids research* (2016). DOI: 10.1093/nar/gkw767.

[4] A. Kiran and P. V. Baranov. DARNED: a DAtabase of RNa EDiting in humans. *Bioinformatics (Oxford, England)* 26.14 (2010), pp. 1772–6. DOI: 10.1093/bioinformatics/btq285.

[5] G. Ramaswami and J. B. Li. RADAR: a rigorously annotated database of A-to-I RNA editing. *Nucleic acids research* 42.Database issue (2014), pp. D109–13. DOI: 10.1093/nar/gkt996.

[6] K. Wang, M. Li, and H. Hakonarson. ANNOVAR: functional annotation of genetic variants from high-throughput sequencing data. *Nucleic acids research* 38.16 (2010), e164. DOI: 10.1093/nar/gkq603.

[7] A. Mortazavi, B. A. Williams, K. McCue, L. Schaeffer, and B. Wold. Mapping and quantifying mammalian transcriptomes by RNA-Seq. *Nature methods* 5.7 (2008), pp. 621–8. DOI: 10.1038/nmeth.1226.

[8] P. Flicek, I. Ahmed, M. R. Amode, D. Barrell, K. Beal, S. Brent, D. Carvalho-Silva, P. Clapham, G. Coates, S. Fairley, S. Fitzgerald, L. Gil, C. García-Girón, L. Gordon, T. Hourlier, S. Hunt, T. Juettemann, A. K. Kähäri, S. Keenan, M. Komorowska, E. Kulesha, I. Longden, T. Maurel, W. M. McLaren, M. Muffato, R. Nag, B. Overduin, M. Pignatelli, B. Pritchard, E. Pritchard, H. S. Riat, G. R. S. Ritchie, M. Ruffier, M. Schuster, D. Sheppard, D. Sobral, K. Taylor, A. Thormann, S. Trevanion, S. White, S. P. Wilder, B. L. Aken, E. Birney, F. Cunningham, I. Dunham, J. Harrow, J. Herrero, T. J. P. Hubbard, N. Johnson, R. Kinsella, A. Parker, G. Spudich, A. Yates, A. Zadissa, and S. M. J. Searle. Ensembl 2013. *Nucleic acids research* 41.Database issue (2013), pp. D48–55. DOI: 10.1093/nar/gks1236.

[9] S. P. Shah, R. D. Morin, J. Khattra, L. Prentice, T. Pugh, A. Burleigh, A. Delaney, K. Gelmon, R. Guliany, J. Senz, C. Steidl, R. A. Holt, S. Jones, M. Sun, G. Leung, R. Moore, T. Severson, G. A. Taylor, A. E. Teschendorff, K. Tse, G. Turashvili, R. Varhol, R. L. Warren, P. Watson, Y. Zhao, C. Caldas, D. Huntsman, M. Hirst, M. A. Marra, and S. Aparicio. Mutational evolution in a lobular breast tumour profiled at single nucleotide resolution. *Nature* 461.7265 (2009), pp. 809–13. DOI: 10.1038/nature08489.

[10] P. Danecek, C. Nellåker, R. E. McIntyre, J. E. Buendia-Buendia, S. Bumpstead, C. P. Ponting, J. Flint, R. Durbin, T. M. Keane, and D. J. Adams. High levels of RNA-editing site conservation amongst 15 laboratory mouse strains. *Genome biology* 13.4 (2012), p. 26. DOI: 10.1186/gb-2012-13-4-r26.

[11] R. Zhang, X. Li, G. Ramaswami, K. S. Smith, G. Turecki, S. B. Montgomery, and J. B. Li. Quantifying RNA allelic ratios by microfluidic multiplex PCR and sequencing. *Nature methods* 11.1 (2014), pp. 51–4. DOI: 10.1038/nmeth.2736.

[12] A. Wright and B. Vissel. The essential role of AMPA receptor GluR2 subunit RNA editing in the normal and diseased brain. *Frontiers in Molecular Neuroscience* 5 (2012), p. 34. DOI: 10.3389/fnmol.2012.00034.

[13] M. Sakurai, H. Ueda, T. Yano, S. Okada, H. Terajima, T. Mitsuyama, A. Toyoda, A. Fujiyama, H. Kawabata, and T. Suzuki. A biochemical landscape of A-to-I RNA editing in the human brain transcriptome. *Genome Research* 24.3 (2014), pp. 522–534. DOI: 10.1101/gr.162537.113.

[14] E. Picardi, A. Gallo, F. Galeano, S. Tomaselli, and G. Pesole. A novel computational strategy to identify A-to-I RNA editing sites by RNA-Seq data: de novo detection in human spinal cord tissue. *PloS one* 7.9 (2012), e44184. DOI: 10.1371/journal.pone.0044184.

[15] T. H. M. Chan, A. Qamra, K. T. Tan, J. Guo, H. Yang, L. Qi, J. S. Lin, V. H. E. Ng, Y. Song, H. Hong, S. T. Tay, Y. Liu, J. Lee, S. Y. Rha, F. Zhu, J. B. Y. So, B. T. Teh, K. G. Yeoh, S. Rozen, D. G. Tenen, P. Tan, and L. Chen. ADAR-Mediated RNA Editing Predicts Progression and Prognosis of Gastric Cancer. *Gastroenterology* (2016). DOI: 10.1053/j.gastro.2016.06.043.

[16] A. Athanasiadis, A. Rich, and S. Maas. Widespread A-to-I RNA editing of Alu-containing mRNAs in the human transcriptome. *PLoS Biology* 2.12 (2004). DOI: 10.1371/journal.pbio.0020391.

[17] C. Quelen, Y. Eloit, C. Noirot, M. Bousquet, and P. Brousset. RNA editing in acute myeloid leukaemia with normal karyotype. *British journal of haematology* 173.5 (2016), pp. 788–90. DOI: 10.1111/bjh.13631.

[18] O. Solomon, L. Bazak, E. Y. Levanon, N. Amariglio, R. Unger, G. Rechavi, and E. Eyal. Characterizing of functional human coding RNA editing from evolutionary, structural, and dynamic perspectives. *Proteins* 82.11 (2014), pp. 3117–31. DOI: 10.1002/prot.24672.

[19] Y. Pinto, H. Y. Cohen, and E. Y. Levanon. Mammalian conserved ADAR targets comprise only a small fragment of the human editosome. *Genome biology* 15.1 (2014), R5. DOI: 10.1186/gb-2014-15-1-r5.

[20] L. Han, L. Diao, S. Yu, X. Xu, J. Li, R. Zhang, Y. Yang, H. M. J. Werner, A. K. Eterovic, Y. Yuan, J. Li, N. Nair, R. Minelli, Y. H. Tsang, L. W. T. Cheung, K. J. Jeong, J. Roszik, Z. Ju, S. E. Woodman, Y. Lu, K. L. Scott, J. B. Li, G. B. Mills, and H. Liang. The Genomic Landscape and Clinical Relevance of A-to-I RNA Editing in Human Cancers. *Cancer Cell* 28.4 (2015), pp. 515–528. DOI: 10.1016/j.ccell.2015.08.013.

[21] L. Kang, X. Liu, Z. Gong, H. Zheng, J. Wang, Y. Li, H. Yang, J. Hardwick, H. Dai, R. T. P. Poon, N. P. Lee, M. Mao, Z. Peng, and R. Chen. Genome-wide identification of RNA editing in hepatocellular carcinoma. *Genomics* 105.2 (2015), pp. 76–82. DOI: 10.1016/j.ygeno.2014.11.005.

[22] X. Hu, S. Wan, Y. Ou, B. Zhou, J. Zhu, X. Yi, Y. Guan, W. Jia, X. Liu, Q. Wang, Y. Qi, Q. Yuan, W. Huang, W. Liao, Y. Wang, Q. Zhang, H. Xiao, X. Chen, and J. Huang. RNA over-editing of BLCAP contributes to hepatocarcinogenesis identified by whole-genome and transcriptome sequencing. *Cancer letters* 357.2 (2015), pp. 510–9. DOI: 10.1016/j.canlet.2014.12.006.
